# Clinical Uncertainty in Large Vessel Occlusion ischemic stroke (CULVO): Does automated perfusion scanning make a difference? Protocol of an intrarater and interrater agreement study

**DOI:** 10.1371/journal.pone.0297520

**Published:** 2024-01-30

**Authors:** Jose Danilo Bengzon Diestro, Robert Fahed, Anass Benomar, Abdelsimar T. Omar, Vitor Mendes Pereira, Julian Spears, Thomas R. Marotta, Pascal Djiadeu, Sunjay Sharma, Forough Farrokhyar

**Affiliations:** 1 Department of Health Research Methods, Evidence, and Impact, McMaster University, Hamilton, Ontario, Canada; 2 Division of Diagnostic and Therapeutic Neuroradiology, Department of Medical Imaging, Unity Health- St Michael’s Hospital, University of Toronto, Toronto, Ontario, Canada; 3 Division of Neurology, Department of Medicine, The Ottawa Hospital, Ottawa, Ontario, Canada; 4 Department of Radiology, Centre Hospitalier de l’Université de Montréal (CHUM), Montreal, Quebec, Canada; 5 Division of Neurosurgery, Department of Surgery, McMaster University, Hamilton, Ontario, Canada; 6 Harvard TH Chan School of Public Health, Harvard University, Boston, Massachusetts, United States of America; 7 Division of Neurosurgery, Department of Surgery, Unity Health- St Michael’s Hospital, University of Toronto, Toronto, Ontario, Canada; 8 Department of Global Health, McMaster University, Hamilton, Ontario, Canada; Kaohsuing Medical University Hospital, TAIWAN

## Abstract

**Background:**

Guidelines recommend the use of perfusion computed tomography (CT) to identify emergent large vessel ischemic stroke (ELVIS) patients who are likely to benefit from endovascular thrombectomy (EVT) if they present within 6–24 hour (late window) of stroke onset. We aim to determine if the interrater and intrarater reliability among physicians when recommending EVT is significantly different when perfusion CT or non-perfusion CT is reviewed.

**Methods:**

A total of 30 non-consecutive patients will be selected from our institutional database comprising 3144 cranial CT scans performed for acute stroke symptoms January 2018 to August 2022. The clinical and radiologic data of the 30 patients will be presented in random order to a group of 29 physicians in two separate sessions at least three weeks apart. In each session, the physicians will evaluate each patient once with automated perfusion images and once without. We will use non-overlapping 95% confidence intervals and difference in agreement classification as criteria to suggest a difference between the Gwet AC1 statistics (κ_G_).

**Discussion:**

The results obtained from this study, combined with the clinical outcomes data of patients categorized through the two imaging techniques and a cost-effectiveness analysis, will offer a comprehensive evaluation of the clinical utility of perfusion CT neuroimaging. Should there be no significant disparity in the reliability of decisions made by clinicians using the two neuroimaging protocols, it may be necessary to revise existing recommendations regarding neuroimaging in the later time window to align with these findings.

## Introduction

### Background of neuroimaging in endovascular thrombectomy

Emergent large vessel ischemic stroke (ELVIS) is a type of stroke caused by the occlusion of major artery supplying the brain with a thrombus or an embolus. Despite best medical management, such occlusions lead to a considerable morbidity, with up to 70–90% of patients being unable to achieve independent living [1, 2]. In 2015, five randomized controlled trials demonstrated the efficacy of endovascular thrombectomy (EVT) in the treatment of emergent large vessel ischemic stroke (ELVIS) involving the anterior circulation of the brain and presenting within 6 hours of symptom onset [3–7].

**Fig 1:**
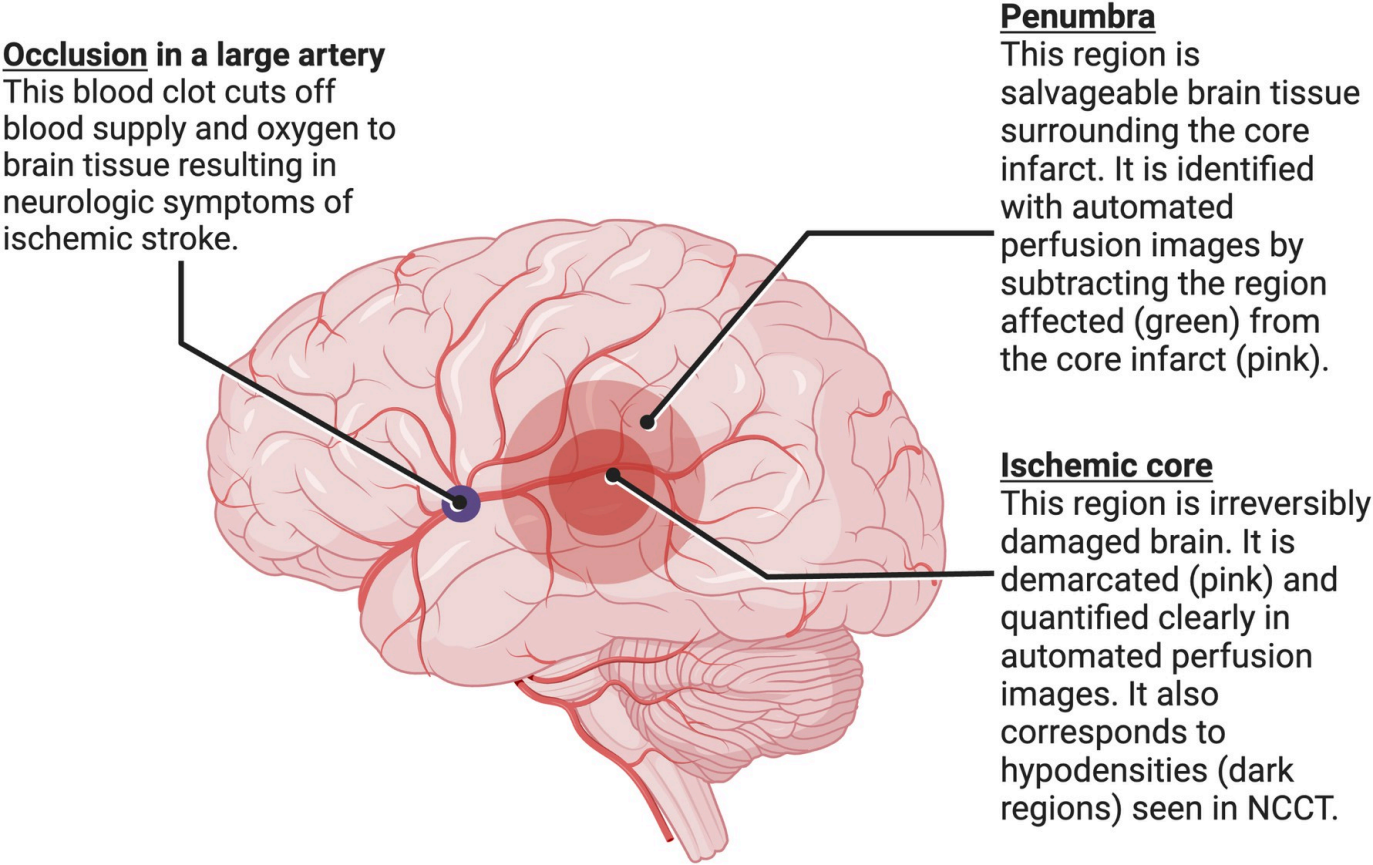
Penumbra and ischemic core. The figure demonstrates the two regions of brain that are affected by an occlusion in a large artery supplying the brain. (Adapted from “Brain Damage (Layout)”, by BioRender.com (2023). Retrieved from https://app.biorender.com/biorender-templates).

EVT involves the manipulation of a stent retriever or aspiration catheter inside a major artery supplying the brain to pull an occlusive blood clot out and restore circulation to the brain. The implementation of EVT for appropriately chosen patients leads to a reduction in ELVIS-related morbidity to a range of 50–70%, varying based on the extent of pre-interventional morbidity [1, 2]. Fig 1 demonstrates the basic rationale using neuroimaging to select patients for the intervention. Patients with larger penumbra (salvageable brain tissue) compared to core infarct (unsalvageable brain tissue) will typically benefit most from the intervention [8]. As time passes, without appropriate intervention, the entire affected region becomes infarcted core.

**Fig 2:**
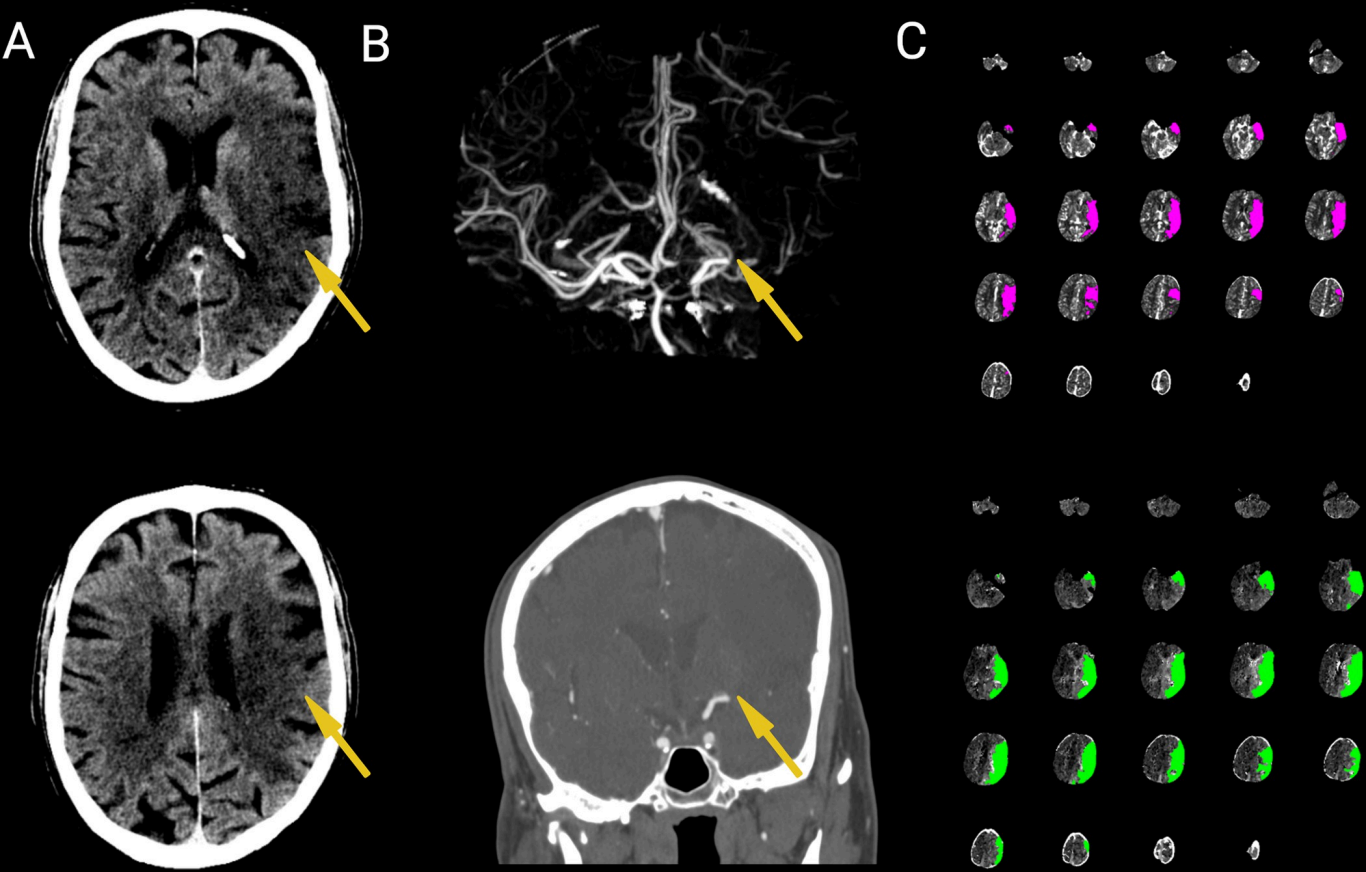
Neuroimaging modalities for acute ischemic stroke. A. Non-contrast, cranial computed tomography (NCCT) scan showing a wedge-shaped hypodensity (yellow arrow) demarcating core infarct (unsalvageable brain) B. Computed tomography angiogram showing occlusion of the left middle cerebral artery C. Automated perfusion scan (RAPID software iSchemaView, Menlo Park, CA) showing the area of core infarct in pink (upper right, 130cc) and the entire area at risk in green (lower right, 209cc) giving an estimated 79cc (209cc-130cc) of penumbra (affected but salvageable brain) (Created with BioRender.com).

Prior to recommending treatment for an ELVIS patient, a non-contrast cranial computed tomography (NCCT) scan, a computed tomography angiogram (CTA) is usually reviewed by a stroke neurologist together with a neurointerventionalist. A CTA is necessary to demonstrate the presence of a clot in the intracranial vasculature. The removal of this occlusion is the target of EVT. It also demonstrates the extent of collateral vasculature that can help supply areas of the brain that have lost arterial blood supply. The extent of collateral circulation has correlated to excellent outcomes in ELVIS patients [9]. An NCCT scan rules out the presence of a large intracranial hemorrhage that may manifest with symptoms similar to an ELVIS. It can also show the extent of core infarct that is already present. Depending on institutional practice and the clinical picture a perfusion CT may also be obtained. Automated perfusion imaging (RAPID, iSchemaView, Menlo Park, CA) gives exact quantitative values of the core infarct and the area of the brain affected by the occlusion. In doing so, the exact area of affected but salvageable brain, the penumbra, is derived by subtracting the two values [10]. (see Fig 2).

The current American Heart Association Guidelines and Canadian Stroke Best Practices recommend the EVT for ELVIS patients presenting within 6 hours of symptom onset or last known well time [11, 12]. Last known well time is defined as the last time a patient was seen at their baseline function prior to the onset of stroke symptoms. This is used a surrogate in patients who have unwitnessed onset of stroke or those who wake up with stroke symptoms already. In this early window period (<6 hours) the decision to perform EVT is based on clinical status and neuroimaging consisting of only a NCCT scan and a CT angiogram (CTA) [11].

For patients coming in the 6–24 hour window from stroke onset, both the American Heart Association guidelines and Canadian Stroke Best Practices recommend the use of perfusion imaging to quantify degree of mismatch and ischemic cores size before deciding on performing EVT [11, 12]. This recommendation is based on two randomized controlled trials, DEFUSE 3 and DAWN, that demonstrated EVT benefit in the at 6–24 hours (late-window) after stroke onset [13, 14]. Both trials exhibit a tendency towards a more explanatory methodology on the explanatory-pragmatic spectrum due to their utilization of stringent inclusion criteria derived from values obtained through automated perfusion imaging. It is imperative to bear in mind that the discrepancy in neuroimaging requisites for the early (<6 hours) and late (6–24 hours) time frames is not predicated on a randomized trial that evaluates the efficacy of the two distinct imaging modalities against each other. Rather, observational data on the utility of perfusion imaging led to its incorporation in the inclusion criteria for the DEFUSE 3 trial [13]. As a result, patients without perfusion imaging could not even be included in this trial.

The use of automated perfusion scanning aims to identify patients who already have large ischemic cores. It is hypothesized that EVT in these patients may either be futile or pose an increased risk of hemorrhagic transformation. However, this assertation has been brought into question with three new randomized controlled trials showing that even ELVIS patients with large cores may still benefit from EVT [2, 15, 16]. Pooled data in this subset of patients, showed that those who undergo EVT with best medical management are 2.34 times more likely to have functional independence compared to those who only receive best medical management [17]. No significant differences in both treatment groups were seen with regards to symptomatic intracranial hemorrhage rate and mortality.

With the new data, we hypothesize that the precise cut-off measurements generated by automated perfusion CT to exclude large ischemic core ELVIS patients may not be as critical as previously thought. Moreover, a recent large retrospective observational study, CLEAR, found that ELVIS patients undergoing EVT utilizing non-perfusion CT imaging for decision-making had comparable clinical outcomes compared to those that utilized perfusion CT or magnetic resonance imaging [18]. Systematic reviews comparing outcomes between late-window ELVIS patients who obtain non-perfusion CT and perfusion CT prior to undergoing EVT do not show a significant difference in benefit in terms of clinical outcomes [19, 20]. However, these reviews are all based on observational data from large databases. An upcoming trial, "A Randomized Trial of Imaging Selection Modalities for Stroke Thrombectomy (NO-CTP)" (NCT05230914), has the potential to provide a more impartial response to the query of whether the preferred imaging modality has any impact on the clinical outcomes of late window ELVIS patients [21].

### What is the problem and how does our study contribute?

Access to the required advanced neuroimaging may pose a challenge for smaller centers and developing countries. Thus, strict adherence to the AHA guidelines and Canadian Stroke Best Practices may even result in ELVIS patients being denied EVT in centers where advanced neuroimaging is not readily available. In addition, the use of automated CT perfusion imaging also poses a significant financial burden—1.3 million CAD in the first year of its implementation in the province of Ontario and an additional 0.9 million CAD each year thereafter [22]. The incorporation of automated perfusion CT may prove to be a suboptimal allocation of resources, if its routine use fails to yield any discernible improvement in clinical outcomes or influence clinical decision-making.

The impact of automated perfusion CT on specialists’ recommendations regarding EVT for late window ELVIS patients was examined to assess its relevance. If perfusion maps do not significantly alter specialists’ decision-making, their practical utility in clinical practice may be questionable. A previous study demonstrated that addition of automated perfusion CT may result in a lower threshold for an EVT recommendation [23]. However, the study had limited representation of physicians and cases as it only involved two raters from the same center and patients were selected consecutively.

**Fig 3:**
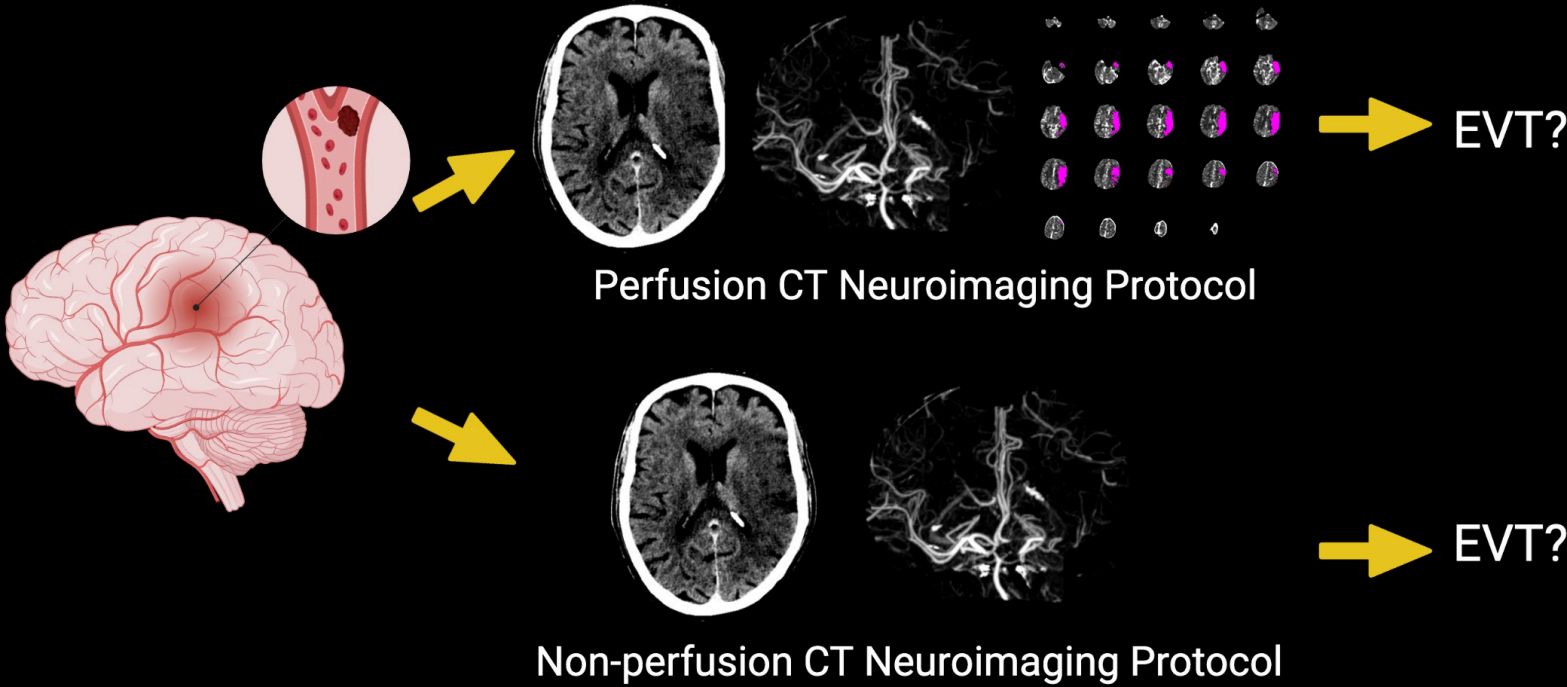
Study protocol. A patient with emergent large vessel ischemic stroke (ELVIS) will be presented to the raters with either of the two neuroimaging protocols: perfusion computed tomography (CT) neuroimaging protocol (non-contrast cranial CT scan (NCCT), computed tomography angiography (CTA), automated perfusion imaging) or non-perfusion CT neuroimaging protocol (NCCT and CTA only). Based on clinical and radiologic details the rater will then decide whether to recommend for or against EVT. (Created with BioRender.com).

By performing an experimental survey on stroke neurologists and neurointerventionalists with cases that encompass the full gamut of ELVIS, we aim to determine whether the use of perfusion CT neuroimaging (non-contrast CT scan, CTA, and automated perfusion scan) demonstrates better reliability of physicians on repeated surveys (intrarater) and among the physicians of different subspecialties and experience (interrater) compared to non-perfusion CT neuroimaging (non-contrast CT scan and CTA). (See Fig 3) Another objective of the study was to examine the impact the types of neuroimaging protocol on raters’ decision-making, specifically assessing whether the presence or absence of automated CT perfusion images and quantitative values led to an increase or decrease in recommendations for EVT.

### Study questions

1. Among stroke neurologists and neurointerventionalists, what is the difference in the proportion of patients recommended for EVT when these patients are evaluated with neuroimaging data from perfusion CT neuroimaging (NCCT, CTA, and automated perfusion imaging) and non-perfusion CT neuroimaging (NCCT, CTA)?
2. Among stroke neurologists and neurointerventionalists is there a significant difference, in their interrater agreement to recommend EVT for late-window ELVIS patients, between cases shown with perfusion CT neuroimaging (NCCT, CTA and automated perfusion imaging) and non-perfusion CT neuroimaging(NCCT, CTA)?
3. Among stroke neurologists and neurointerventionalists is there a significant difference, in their intra-rater agreement to recommend EVT for late-window ELVIS patients, between cases shown with perfusion CT neuroimaging (NCCT, CTA and automated perfusion imaging) and non-perfusion CT neuroimaging(NCCT, CTA)?

## Methodology

### Agreement study

The study will be prepared in accordance with the Guidelines for Reporting Reliability and Agreement Studies (GRRAS) [24]. The guideline was made primarily to evaluate reliability in ascertaining parameters based on a particular diagnostic modality. We adopted a novel approach in our research methodology by incorporating agreement statistics to evaluate clinical decision-making, which was inspired by a recently published innovative study [25]. Instead of making a diagnosis we sought to evaluate whether the raters agreed on a recommendation for EVT given clinical and radiologic characteristics of the cases presented to them.

### Case selection

All included studies will be taken from an institutional database (January 2018- August 2022) of patients undergoing in-house neuroimaging for symptoms of acute ischemic stroke. A team of clinical and research physicians will review the entire database to identify reported large vessel occlusions in patients being scanned for symptoms of stroke from January 2018 to August 2022. The primary author who is a dual trained stroke neurologists and neurointerventionalist will review all the patients that were flagged to determine eligibility for the study. The inclusion criteria will include the following:

1. Adults aged ≥ 18 years old
2. Stroke onset or last known well time between 6–24 hours prior to start of imaging (late-window stroke)
3. Use of automated perfusion CT (RAPID, iSchemaView, Menlo Park, CA)
4. Confirmed complete large vessel occlusion on CTA involving the following vessels: first segment of the middle cerebral artery (M1), terminal portion of the internal carotid artery (ICA), tandem occlusion of the proximal ICA and either of the occlusions (M1 and terminal ICA)
5. Penumbra size of at least 15cc of perfusion CT
6. Mismatch ratio (volume of the entire area affected: core infarct volume) of at least 1.8
7. National Institutes of Health Stroke Score (NIHSS) of 6 or more

Our exclusion criteria were:

1. Neuroimaging findings reported to be compatible with a chronic occlusion
2. Occlusions located in the distal anterior circulation territory or the posterior circulation
3. Missing clinical data
4. Unsatisfactory neuroimaging (artifacts, improper timing of contrast, poor quality perfusion studies)

To minimize the paradoxes of Kappa statistics [26, 27], we will include the full range of infarct core sizes: 10 cases with small core (0–49 cc), 10 cases with medium core (50-100cc), 10 cases with large core (>100cc). Sequential, consecutive inclusion of cases will result in an imbalanced distribution as cases of small to moderate cores will likely dominate the selection because the institution is a tertiary referral center that will preferentially transfer those with smaller cores. Evidence for EVT in ELVIS patients with large core is a recent development; thus, all the patients were transferred to our institution during a time when the only positive randomized EVT trials were for small to medium core patients. This would lead to a paradox of high percent agreement (only by chance) but low reliability [26, 27].

The selection process will be guided by the aim of maximizing the representation of diverse clinical and radiologic features, allowing for a comprehensive assessment of the reliability of neuroimaging protocols in real-world scenarios. We reiterate that the spectrum of patients that we chose is not an attempt to replicate the frequencies and proportions that are seen in our daily practice—a predominance of smaller infarct core patients. Doing so may result in falsely elevated agreement statistics. Apart from minimizing the paradoxes of the Kappa statistic, we would like to determine the reliability (repeatability) of clinician decisions in an experimental setting that accommodates the full spectrum of core infarct sizes [25]. We expect that raters would exhibit a preference for recommending EVT for patients with small core infarctions, advise against it for patients with large core infarctions, and consider patients with medium core infarctions as falling within a subjective and uncertain range. We purposely included extremes in value for other variables too such as age and NIHSS to really test whether physicians decisions are reliable across a wide spectrum of cases.

The rationale for the three main divisions of core sizes is based on previous studies on EVT for ELVIS. In the DEFUSE 3 trial that demonstrated the benefit of EVT for late window ELVIS patients the great majority of the patients randomized had core infarct sizes less than 50cc—median core infarct volumes were around 9-10cc [13]. Thus, we considered these patients to have a small core. Moderate sized cores were sized at 50–100 cc consistent with a then planned randomized trial for large core ELVIS patients [28]. Core infarcts that were larger than this size were considered large cores infarcts. The size of core infarcts represents the areas of the brain deemed unsalvageable. Intuitively, clinicians would be more inclined to treat patients with smaller core sizes. We also only included cases with a significant penumbra characterized as salvageable brain tissue measuring at least 15 cc with a mismatch ratio of 1.8 or more as defined by the DEFUSE 3 study [13]. The mismatch ratio is calculated by dividing the volume of the total area affected (penumbra and core infarct) over the core infarct (unsalvageable tissue). (see Fig 2)

### Survey

Cases will be collected and managed using Microsoft Excel v16.72 and REDCap (Research Electronic Data Capture) electronic data capture tools hosted at the Unity Health- St. Michael’s Hospital. REDCap is a secure, web-based software platform designed to support data capture for research studies, providing 1) an intuitive interface for validated data capture; 2) audit trails for tracking data manipulation and export procedures; 3) automated export procedures for seamless data downloads to common statistical packages; and 4) procedures for data integration and interoperability with external sources [29, 30]. Individual, personalized links were sent to the raters via email. Raters were asked details about their practice: type of hospital affiliation, specialty, years in practice and institutional neuroimaging practices for stroke patients. The full survey is in **S1 Appendix.**

For each of the selected 30 patients the following details will be provided: age, location of occlusion, the NIHSS (National Institutes of Health Stroke Score), time of onset, time of scan, non-perfusion CT neuroimaging (NCCT and CTA). The NCCT scan will be shown in the recommended stroke window settings (35WW 35LL) [31–33]. Doing so ensures that the raters are able to evaluate the scan in a window that is optimized for showing the early ischemic changes important for ASPECTS.

The same 30 cases will be shown again with the same information but with perfusion CT neuroimaging (NCCT, CTA and automated perfusion imaging). Overall, the raters will encounter 60 cases comprised of the same 30 patients shown twice with and without perfusion imaging. The raters will not be informed that these 60 cases are actually the same 30 patients shown twice. To test intra-rater agreement, a second round of rating will be done at least 3 weeks from the submission of the initial reading [34]. The order in which the cases are shown will be the same for the second rating. Apart from not telling the raters, the following measures were also taken to keep the raters realizing that the same 30 cases are being shown:

1. Invitation e-mails will have an abbreviated title, “Clinical uncertainty in large vessel occlusion ischemic stroke: An intrarater and interrater agreement study”, instead of, “Clinical uncertainty in large vessel occlusion ischemic stroke: Does automated perfusion scanning make a difference? An intrarater and interrater agreement study”
2. Each case will be presented separately in a single screen. The back button for the survey was disabled; consequently, raters were unable to review their previous answers.
3. A random number generator will be used to determine the order all the cases.

For each case, the raters will be asked to grade the ASPECTS, the single-phase collateral score (collateral score 0: absence of vessels distal to the occlusion, 1: ≤ 50% but >0% collateral supply, 2: ≥ 50% but <100% collateral supply or 3: 100% collateral supply), and finally whether they would recommend EVT based on the available clinical and radiologic data [9, 35]. The ASPECTS (0–10) divides the middle cerebral artery territory into 10 areas and takes a point away for each area that appears to have early ischemic changes. Prior to answering questions pertaining to 60 cases, a short review of the ASPECTS and single-phase CT collateral score was done. **(see S1 Appendix)**

Prior to inviting official study raters, the final version of the survey will be sent to 5–10 physicians who are trainees in the field of stroke neurology and neurointervention. Edits will be made to the survey based on their feedback prior to starting the study with the official raters. The trainees who will participate in the pilot testing are those that do not meet the study’s rater inclusion criteria.

### Imaging acquisition

All scans were performed using a 256‐slice GE Revolution volumetric CT scanner (GE Medical Systems, Madison, USA). An axial non-contrast CT (NCCT) was first obtained, followed by a timing bolus with region of interest (ROI) placed in the internal carotid artery at the C2-C3 level to optimize multiphase CT angiography (mCTA) acquisition; mCTA was then performed, with a tube voltage of 120 kV, organ-modulated tube current ranging from 150–475 mA, section thickness of 0.625 mm, and reconstruction at 4 mm thickness. A test bolus of 20 ml of iodinated contrast material (iohexol 350 mg I/ml, Omnipaque, GE Healthcare Co., Ltd., Shanghai, China) was injected intravenously, followed by 20 ml of saline flush at a rate of 5 ml/s, while 65 ml of iodinated contrast material followed by 30 ml of saline was also administered at a rate of 5 ml/s for the mCTA using power injection. The first phase covered the aortic arch to the skull vertex, and was obtained 12 seconds after a delay determined from the timing bolus. Subsequently, a second phase covering C3 to the vertex and a third phase covering the skull base to the vertex were acquired after additional delays of 7 and 12 seconds, respectively.

For CT perfusion (CTP), 37 consecutive acquisitions were performed, with a temporal resolution of 1.3 seconds, tube voltage of 80 kV, tube current of 225 mAs, gantry rotation time of 0.5 seconds, coverage in the z-axis of 120 mm, and slice thickness of 5 mm. Forty mL of iodinated contrast was injected at a rate of 4 mL/s, followed by 40 mL of saline flush at 4 mL/s. All CTP images were automatically post-processed using RAPID software (iSchemaView, Menlo Park, California, USA). A relative cerebral blood flow (CBF) threshold of 30% was used to distinguish penumbra from infarct, while a Tmax >6 seconds was used to identify the total ischemic area. Penumbra and infarct volumes were quantified within the entire scan range.

### Rater selection

We will invite physicians who are practicing stroke neurologists and neurointerventionalists in Canada who work in comprehensive stroke centers with access to perfusion imaging. These specialties will be chosen because in the typical clinical pathway these physicians are ultimately responsible and decide whether an ELVIS patient undergoes EVT. While diagnostic neuroradiologists with no neurointervention training are certainly a vital part of the stroke team, they are limited to interpreting imaging or making suggestions for patient management. Invitations will be sent to the physicians through the Canadian Stroke Consortium and the Canadian Interventional Neuro Group. By tapping into these national societies, we hope to include a group of raters with diverse background and differing levels of experience from different parts of the country. The inclusion criteria for the raters will be as follows:

1. Currently practicing in Canada as a stroke neurologist or neurointerventionalist
2. Based out of a comprehensive center that offers EVT
3. Having completed at least 1 year of fellowship in stroke neurology or neurointervention

Clinical fellows will be allowed to participate in the study if they had already finished at least a year of fellowship. These individuals are considered to be junior attendings. While no monetary incentive was provided, the invitation will state that all those who finish both rounds of the survey will be given the opportunity to be a co-author in future publications if they wish. All those with equal to or more than 10 years of experience (post-residency training) will be considered senior raters—the rest will be junior raters. Neurointervenitonalists are physicians who are trained in performing EVT. Stroke neurologists, neuroradiologists and neurosurgeons can all undertake fellowship training to become neurointervenitonalists. Our raters will be classified as: stroke neurologists, neurointerventionalists (neuroradiologists and neurosurgeons) and dual-trained neurologists who are both stroke neurologists and neurointerventionalists. After the study, all raters will be sent a debriefing letter to explain the deception involved in the research. They will all be given a chance to withdraw from the study.

### Sample size

We estimated, using kappaSize package in R version 4.2.0 (R Foundation for Statistical Computing, Vienna, Austria), assuming an anticipated kappa value of 0.6 (substantial), that at least 30 cases were necessary for the lower limit of a 95% two–sided confidence interval to remain above 0.45 between at least 6 raters, considering an anticipated prevalence of EVT recommendation of 0.5 [36]. A rule of thumb for agreement studies with binary outcomes is to have at least 10 raters reviewing at least 30–50 patients [25]. While more patients will enable us to provide an even wider spectrum of patients we also want to avoid rater fatigue. For this study we had 60 cases with three questions each. We need to have at least 6 raters based on our sample size computation but will be aiming for at least 10 to follow the current convention.

### Descriptive statistics

We will summarize patient and rater characteristics. Nominal variables will be presented as frequencies and percentages while the measures of central tendency and dispersion will be used for continuous variables. Median, minimum, and maximum values will be used for continuous variables that are normally distributed by visualization and the Shapiro-Wilk normality test; otherwise, mean and standard deviation values will be used. We will present the difference in EVT recommendations according to the neuroimaging presented to the raters—perfusion CT neuroimaging or non-perfusion CT neuroimaging. Graphs and charts will be done on Microsoft Excel v16.72. We will use the R statistical computing language (R Core Team, 2022) in the RStudio framework to perform all data visualization and descriptive statistics. Specifically, we will utilize the following R packages, including dplyr, readxl, tidyverse, ggplot2, table1 and skimr [37, 38].

### Agreement statistics

The inter-rater and intra-rater agreement of the recommendation of EVT will be assessed using Gwet’s AC1 (κ_G_) reliability coefficient. AC1 stands for agreement coefficient, first order chance correction and is used for binary data [39]. We will derive the κ_G_ for binary (EVT recommendations) data, with 95% bias-corrected confidence intervals. Poor (κ_G_ <0), slight (κ_G_ = 0–0.20), fair (κ_G_ = 0.21–0.40), moderate (κ_G_ = 0.41–0.60), substantial (κ_G_ = 0.61–0.80) and excellent (κ_G_ >0.80) agreement categories were defined according to Landis and Koch and used to classify the K_G_ [40]. Non-overlapping confidence intervals along with level of agreement will be the factors considered to suggest a potential difference between KG values [41]. Reliability calculations will be done according to the following prespecified rater subgroups for interrater reliability: rater specialty, rater experience, infarct core size and time since stoke onset. For intrarater reliability we will look at rater specialties. We will use the R statistical computing language (R Core Team, 2022) in the RStudio framework to compute for the K_G_ values using the irrCAC package [42].

Classically, the Kappa statistic was used to quantify the degree of reliability between raters [43]. Cohen’s Kappa was computed by the following equation: (observed agreement-expected agreement)/ (1-expected agreement). The Kappa paradox arises in situations where the prevalence of the “condition” is high such that the likelihood of agreeing to diagnose the presence of absence of a disease (or recommend EVT as in our study) between raters is very high or very low. In this cirumstance the kappa statistic is disproportionately low despite a high observed agreement [26]. This was demonstrated in a similar clinical decision making agreement study on aneurysm treatment where multiple intrarater kappa values demonstrated paradoxical low kappa values despite high observed agreement [44].

The κ_G_ statistic that was introduced in 2001 has been shown to be relatively resistant to the Kappa paradox and results in values that are more in keeping with the observed agreeement. This is because it has a less severe correction for chance agreement compared to Cohen’s Kappa [39, 45]. As an example, in our study we will look at subgroups according to infarct cores. We expect that in these situations, decisions will be lopsided in that raters will have a much higher tendency to recommend for EVT for small cores and against it for large cores. We chose to use Gwet’s AC1 statistic for our study as we expect it to be a more robust measure of reliability [43].

### Ethics

The study was approved by the research ethics board of Unity Health- St. Michael’s Hospital (SMH REB# 22–186).

## Discussion

Findings of this study, together with data on the clinical outcomes of patients triaged using the two imaging modalities and cost effectiveness analysis should provide a complete assessment of the utility of perfusion CT neuroimaging. If the reliability of decisions made by clinicians is not significantly different between the two neuroimaging protocols, then recommendations regarding neuroimaging in the late time window may need to be modified to reflect these results. Our goal is to determine whether the automated perfusion CT images and quantitative values found in the perfusion CT neurimaging protocol add value to clinical decision making from a reliability standpoint.

Our study is limited to CT neuroimaging and will not tackle cranial MRI—a different imaging modality that may also be used in acute stroke. This will limit the findings of our study as perfusion imaging can also be used with cranial MRIs. Our results will only be applicable to Canadian practice as all our raters are from comprehensive academic centers in this country. As a previous study demonstrated, decision-making is different across the globe even with cases that fit high level recommendations (Level 1A and Level 2B) [46]. Our study also involves some degree of deception of our recruited raters. While we have taken several measures to keep the mechanics of the study confidential, they may still figure out that they are viewing the same cases twice each round. This may lead to efforts towards delivering consistent answers.

Knowledge dissemination will be through presentation in conferences dealing with stroke and neurointervention. Afterwards, the results will be published in peer-reviewed journals.

## Supporting information

S1 Appendix CULVO survey. (PDF)

S1 File CULVO REB approval. (PDF)

S2 File GRAAS agreement checklist CULVO. (PDF)

S3 File Image publication license. (ZIP)
