# Supplementary material for: Clinical Uncertainty in Large Vessel Occlusion ischemic stroke (CULVO): Does automated perfusion scanning make a difference? Protocol of an intrarater and interrater agreement study
Source: PLoS One. 2024 Jan 30;19(1):e0297520. doi: 10.1371/journal.pone.0297520 (PMC10826946; doi:10.1371/journal.pone.0297520)
Supplement: S1 Appendix — (PDF) [file pone.0297520.s001.pdf]

Clinical Uncertainty in Large Vessel Occlusion (CULVO) Study

Dear rater,

Thank you for agreeing to participate in this study entitled, "Clinical Uncertainty in Large Vessel Occlusion ischemic stroke: An intrarater and interrater agreement study".

For each case please indicate the ASPECTS, single phase CT collateral score, and whether or not you recommend EVT (endovascular thrombectomy) for each of these patients based on the available imaging and clinical data. There are 60 cases in all.

Some important points to remember for the activity:

1. There are no right or wrong answers.

2. We recommend spending no more than 2 minutes per case as one usually does in an acute situation. The whole survey should only take about 2 hours.

3. All the cases are in the delayed time window, that is, patients presenting more than 6 hours after their last known well time.

4. The non-contrast CT has been optimized (recommended windowing) for ASPECTS reading.

5. The automated perfusion scan results have all been quality-checked prior to case selection.

6. Though we recommend that you accomplish this in one sitting, you may leave and go back to the study after each case.

7. You will be asked to provide details about your practice profile (experience, institution, specialty, routine use of perfusion imaging).

8. We will provide links and files to refresh your knowledge of ASPECTS and the single-phase collateral score.

9. All your decisions for each case are final. You cannot go back to the previous case once you have decided to recommend or withhold EVT.

All the best,

CULVO Study team

Participant Details

Please enter your email address.

____

1 What institution are you mainly affiliated with affiliated with?

____
(If you are affiliated with both a university and a hospital, please write both.)

Would you consider yourself to have an academic practice, in that you formally pursue academic endeavors as part of your affiliation with a university?

◯ Yes
◯ No

What university or academic institution are you affiliated with?

____

What is your base specialty and training?	◯ Neurology<br>◯ Radiology (Interventional Neuroradiology)<br>◯ Neurosurgery (Endovascular Neurosurgery)<br>◯ I have more than one base specialty. (Selecting this means you have successfully finished at least two residency programs from the 3 choices above)
If you are a neurologist, how would you characterize your current practice as it relates to acute stroke care?	◯ Stroke Neurology only<br>◯ Interventional Neurology only<br>◯ Combined Stroke Neurology and Interventional Neurology
What is your current level of training or practice?	◯ Fellow Stroke/ Cerebrovascular Diseases<br>◯ Fellow Interventional neuroradiology, Endovascular neurosurgery, Interventional neurology<br>◯ Attending Neurologist with practice in acute medical stroke care<br>◯ Attending Neurologist with practice in acute medical stroke care and interventional neurology<br>◯ Attending Interventional neuroradiology, Endovascular neurosurgery, and interventional neurology (for neurologists only choose this option if you no longer do acute medical stroke care, otherwise select option 3)
How many years have you been in practice as it relates to acute stroke care after residency graduation? This number includes fellowship training.	__________________________
In your practice what percentage of late window (> 6 hours after last known well time) is imaged using CT (vs MRI)?	0 50 100<br>*(Place a mark on the scale above)*
Do you have access to perfusion imaging in your practice?	◯ Yes<br>◯ No
Among late window stroke patients (>6 hours after last seen well), what percentage obtain perfusion imaging in your practice?	0 50 100<br>*(Place a mark on the scale above)*
In your practice, do you have access to automated perfusion imaging (RAPID or equivalent software) as part of your acute stroke protocol imaging for late window patients (> 6 hours)?	◯ Yes<br>◯ No
What percentage of late window patients (>6 hours after last seen well) obtain automated perfusion imaging (RAPID or equivalent) in your practice?	0 50 100<br>*(Place a mark on the scale above)*

ASPECTS and Single Phase Collateral Score

Alberta Stroke Program Early CT Score (ASPECTS)

Here is a refresher image: (Case courtesy of Dr. Osamah A. A. Alwalid, Radiopaedia.org, rID: 72706)

Should you need a more in-depth tutorial on the topic please see proceed to the University of Calgary ASPECTS in Acute Stroke website by copying the following link on your address bar:
http://aspectsinstroke.com/ed/course-12/course-5

MCA Alberta stroke program early CT score (ASPECTS)

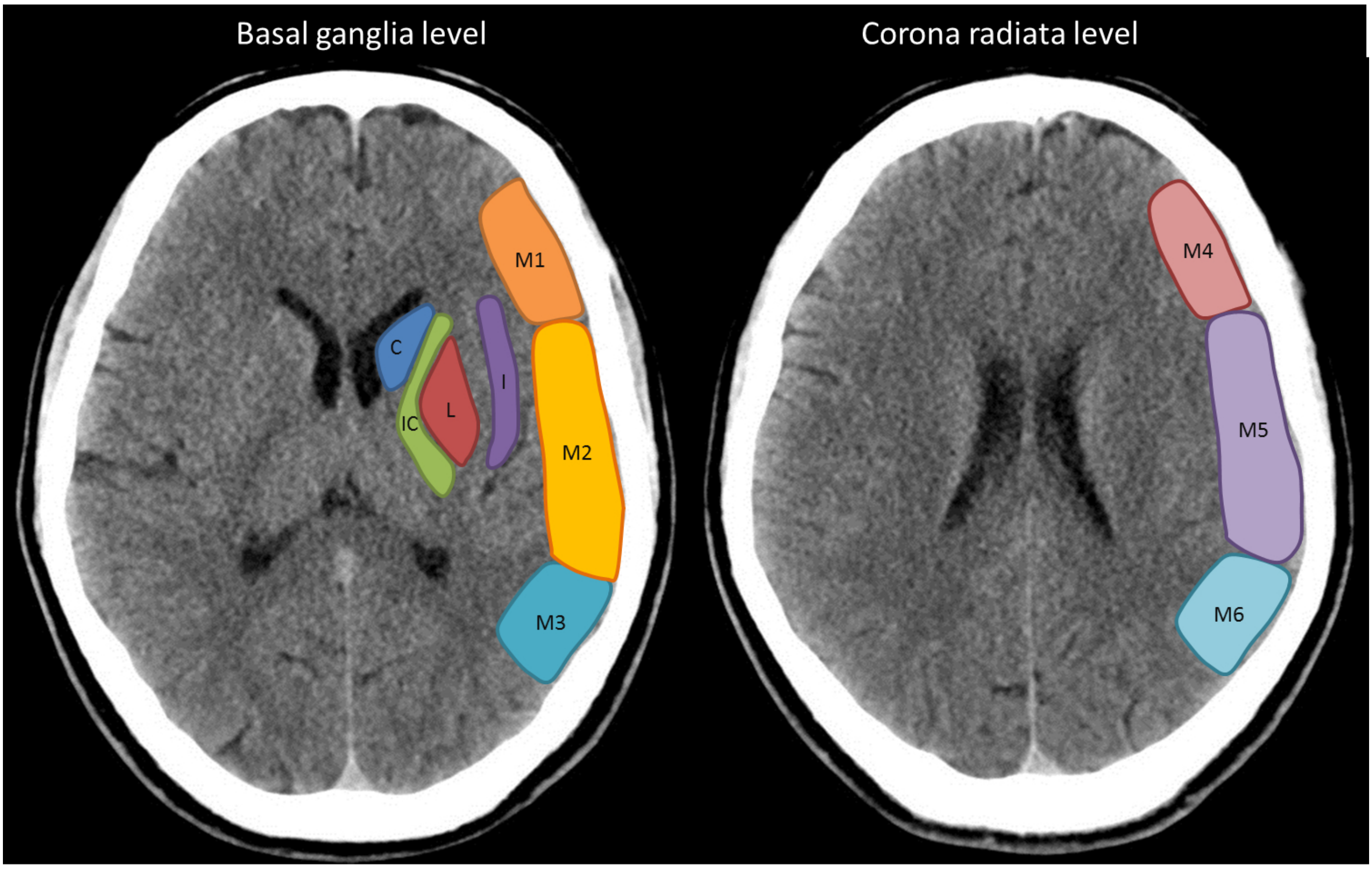

C: Caudate; IC: internal capsule; L: lentiform nucleus; I: Insular Cortex.

Osamah Alwalid
cc BY NC SA
Radiopaedia.org

Single Phase Collateral Score

The following image is adapted from the single-phase collateral score by Tan et al. (CT Angiography Clot Burden Score and Collateral Score: Correlation with Clinical and Radiologic Outcomes in Acute Middle Cerebral Artery Infarct)

Collateral Score (CS) 0: The absence of vessels on CTA distal to the occlusion
Collateral Score (CS) 1: Collateral supply filling < or equal to 50% but >0% of the occluded MCA territory.
Collateral Score (CS) 2: Collateral supply filling >50% but < 100% of the occluded MCA territory
Collateral Score (CS) 3: 100% collateral supply of the occluded MCA territory

The full text of the publication is also available: http://www.ajnr.org/content/30/3/525

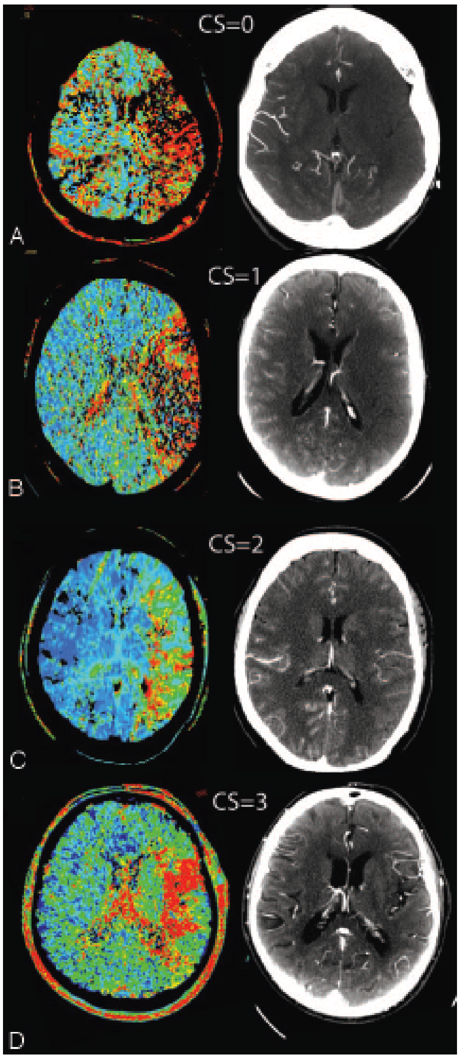

Case 1: 21

Case Details
Demographics 84-year-old male
Occlusion Left M1 occlusion
NIHSS 11
Last known well time 02:00
Time of CT scout film (Start of CT) 17:29

Non-contrast CT Scan

What is the ASPECTS of this scan? ________________________________

Single phase CTA

What is the single-phase collateral score for this scan?

- ◯ Collateral Score 0: Absence of vessels on CTA distal to the occlusion
- ◯ Collateral Score 1: Collateral supply filling < or equal to 50% but >0% of the occluded MCA territory.
- ◯ Collateral Score 2: Collateral supply filling >50% but < 100% of the occluded MCA territory
- ◯ Collateral Score 3: 100% collateral supply of the occluded MCA territory

Perfusion scan

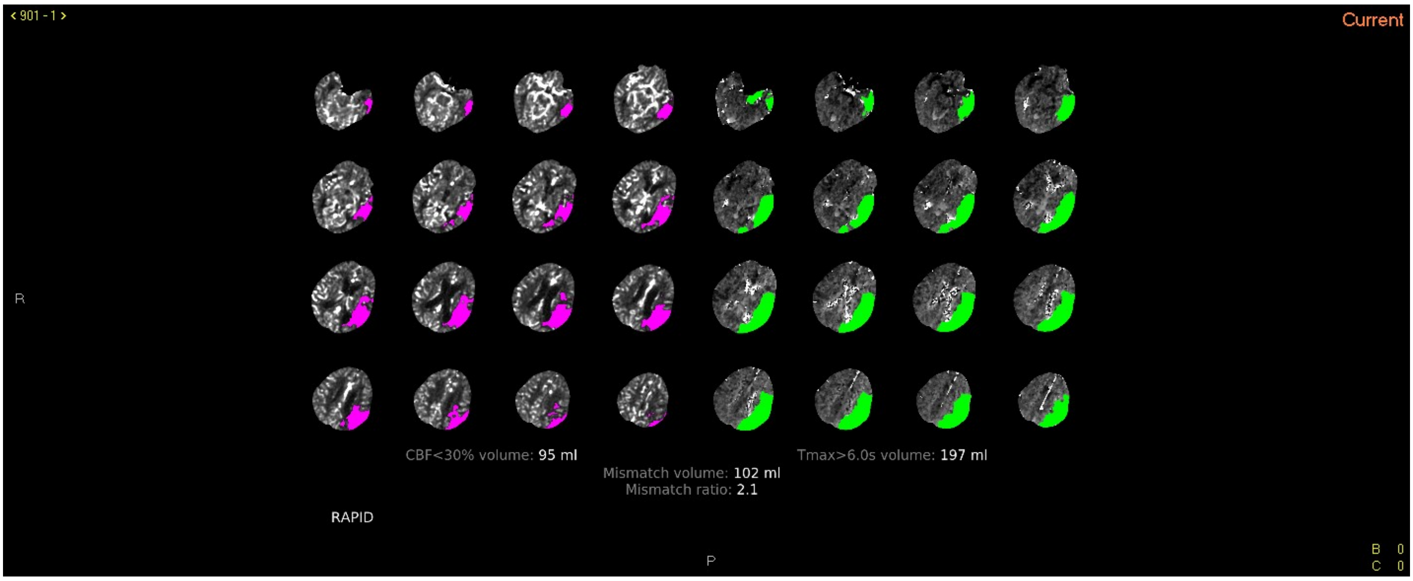

Based on the clinical and radiologic details will you recommend endovascular thrombectomy for this patient?

- ◯ Yes
- ◯ No

Case 2: 32

Case Details
Demographics 74-year-old male
Occlusion Right M1 occlusion
NIHSS 22
Last known well time 12:00 the day prior
Time of CT scout film (Start of CT) 02:38 the following day

Non-contrast CT

What is the ASPECTS of this scan? ______________________

Single Phase CTA

What is the single-phase collateral score for this scan?

- ◯ Collateral Score 0: Absence of vessels on CTA distal to the occlusion
- ◯ Collateral Score 1: Collateral supply filling < or equal to 50% but >0% of the occluded MCA territory.
- ◯ Collateral Score 2: Collateral supply filling >50% but < 100% of the occluded MCA territory
- ◯ Collateral Score 3: 100% collateral supply of the occluded MCA territory

Based on the clinical and radiologic details will you recommend endovascular thrombectomy for this patient?

- ◯ Yes
- ◯ No

Case 3: 44

Case Details
Demographics 74-year-old male
Occlusion Right M1
NIHSS 20
Last known well time 19:00 the day prior
Time of CT scout film (Start of CT) 16:04 the following day

Non-contrast CT

What is the ASPECTS of this scan?

____

Single Phase CTA

What is the single-phase collateral score for this scan?

- ◯ Collateral Score 0: Absence of vessels on CTA distal to the occlusion
- ◯ Collateral Score 1: Collateral supply filling < or equal to 50% but >0% of the occluded MCA territory.
- ◯ Collateral Score 2: Collateral supply filling >50% but < 100% of the occluded MCA territory
- ◯ Collateral Score 3: 100% collateral supply of the occluded MCA territory

Based on the clinical and radiologic details will you recommend endovascular thrombectomy for this patient?

- ◯ Yes
- ◯ No

Case 4: 16

Case Details
Demographics 83-year-old male
Occlusion Right M1 occlusion
NIHSS 15
Last known well time 13:30
Time of CT scout film (Start of CT) 20:39

Non-contrast CT

What is the ASPECTS of this scan? ______________________

Single Phase CTA

What is the single-phase collateral score for this scan?

- ◯ Collateral Score 0: Absence of vessels on CTA distal to the occlusion
- ◯ Collateral Score 1: Collateral supply filling < or equal to 50% but >0% of the occluded MCA territory.
- ◯ Collateral Score 2: Collateral supply filling >50% but < 100% of the occluded MCA territory
- ◯ Collateral Score 3: 100% collateral supply of the occluded MCA territory

Based on the clinical and radiologic details will you recommend endovascular thrombectomy for this patient?

- ◯ Yes
- ◯ No

Case 5: 31

Case Details
Demographics 74-year-old male
Occlusion Right M1 occlusion
NIHSS 22
Last known well time 12:00 the day prior
Time of CT scout film (Start of CT) 02:38 the following day

Non-contrast CT

What is the ASPECTS of this scan? __________________________________

Single Phase CTA

What is the single-phase collateral score for this scan?

- ◯ Collateral Score 0: Absence of vessels on CTA distal to the occlusion
- ◯ Collateral Score 1: Collateral supply filling < or equal to 50% but >0% of the occluded MCA territory.
- ◯ Collateral Score 2: Collateral supply filling >50% but < 100% of the occluded MCA territory
- ◯ Collateral Score 3: 100% collateral supply of the occluded MCA territory

Automated Perfusion Scan Results

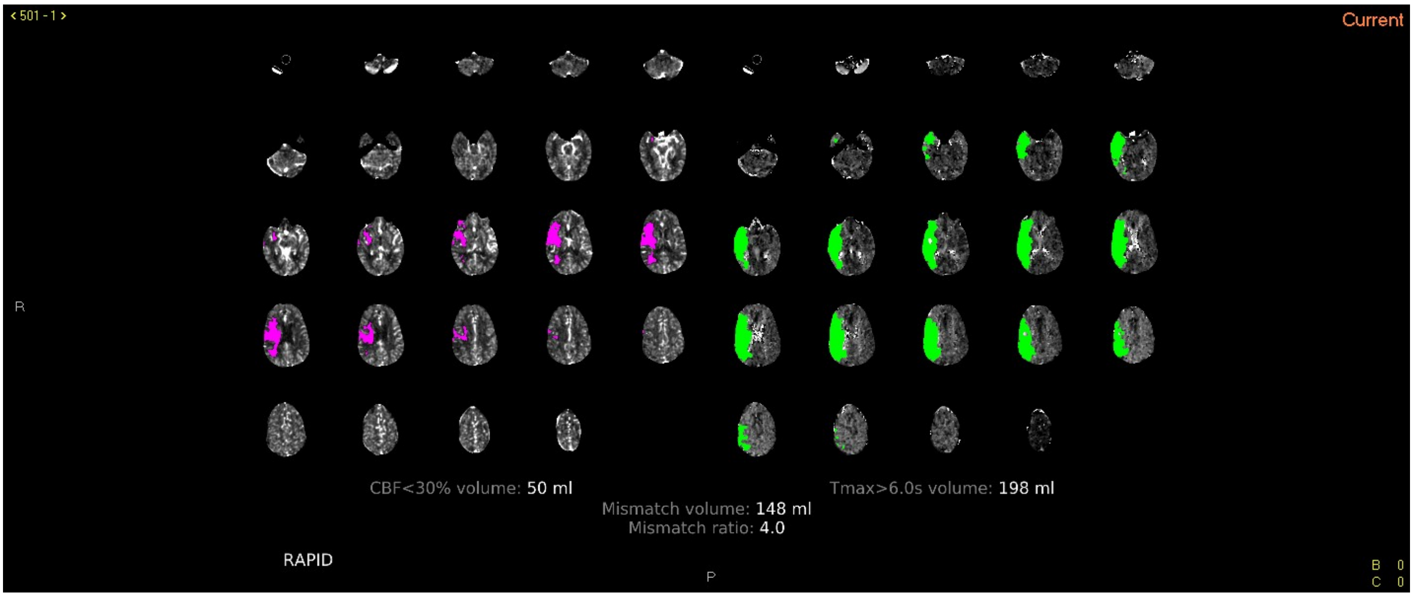

Based on the clinical and radiologic details will you recommend endovascular thrombectomy for this patient?

- ◯ Yes
- ◯ No

Case 6: 13

Case Details
Demographics 79-year-old female
Occlusion Left carotid termination
NIHSS 24
Last known well time 04:30
Time of CT scout film (Start of CT) 11:27

Non-contrast CT

What is the ASPECTS of this scan? ____________________

Single Phase CTA

What is the single-phase collateral score for this scan?

- ◯ Collateral Score 0: Absence of vessels on CTA distal to the occlusion
- ◯ Collateral Score 1: Collateral supply filling < or equal to 50% but >0% of the occluded MCA territory.
- ◯ Collateral Score 2: Collateral supply filling >50% but < 100% of the occluded MCA territory
- ◯ Collateral Score 3: 100% collateral supply of the occluded MCA territory

Automated Perfusion Scan Results

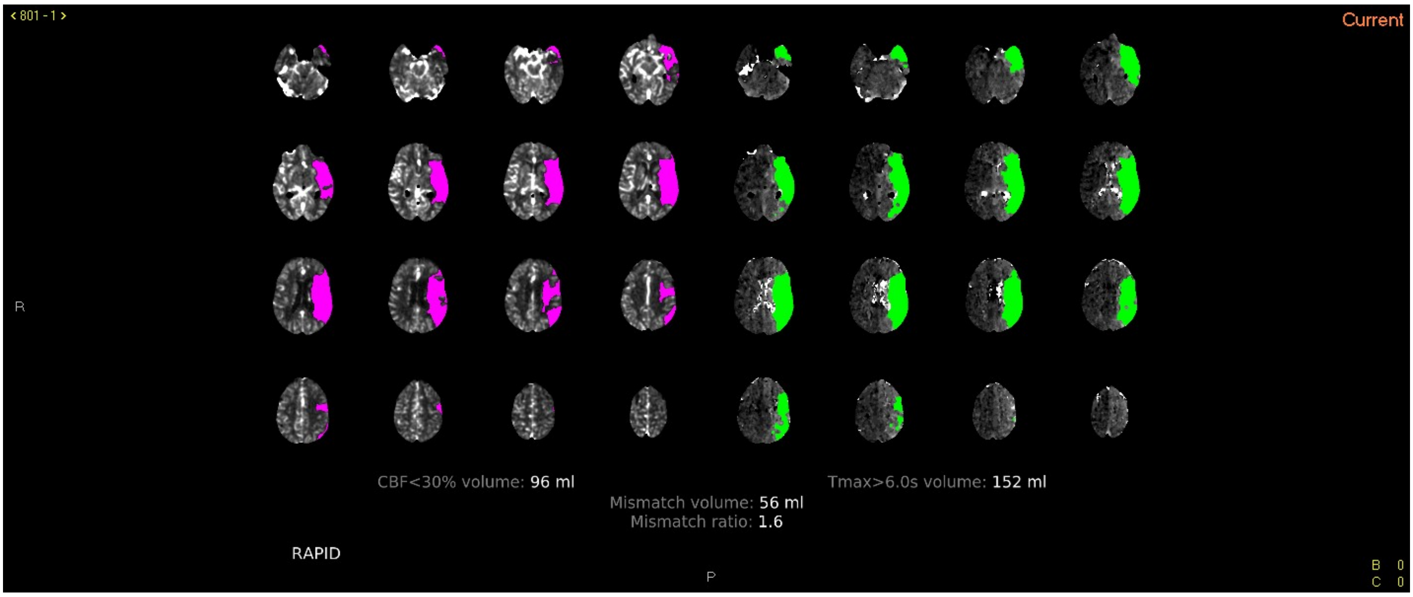

Based on the clinical and radiologic details will you recommend endovascular thrombectomy for this patient?

- ◯ Yes
- ◯ No

Case 7: 58

Case Details
Demographics 71-year-old male
Occlusion Right M1
NIHSS 19
Last known well time 02:30
Time of CT scout film (Start of CT) 11:23

Non-contrast CT

What is the ASPECTS of this scan? ______________________

Single Phase CTA

What is the single-phase collateral score for this scan?

- ◯ Collateral Score 0: Absence of vessels on CTA distal to the occlusion
- ◯ Collateral Score 1: Collateral supply filling < or equal to 50% but >0% of the occluded MCA territory.
- ◯ Collateral Score 2: Collateral supply filling >50% but < 100% of the occluded MCA territory
- ◯ Collateral Score 3: 100% collateral supply of the occluded MCA territory

Based on the clinical and radiologic details will you recommend endovascular thrombectomy for this patient?

- ◯ Yes
- ◯ No

Case 8: 59

Case Details
Demographics 74-year-old male
Occlusion Left carotid termination
NIHSS 22
Last known well time 23:00 The night prior
Time of CT scout film (Start of CT) 09:47 of the current day

Non-contrast CT

What is the ASPECTS of this scan?

____

Single Phase CTA

What is the single-phase collateral score for this scan?

- ◯ Collateral Score 0: Absence of vessels on CTA distal to the occlusion
- ◯ Collateral Score 1: Collateral supply filling < or equal to 50% but >0% of the occluded MCA territory.
- ◯ Collateral Score 2: Collateral supply filling >50% but < 100% of the occluded MCA territory
- ◯ Collateral Score 3: 100% collateral supply of the occluded MCA territory

Automate Perfusion Scan Results

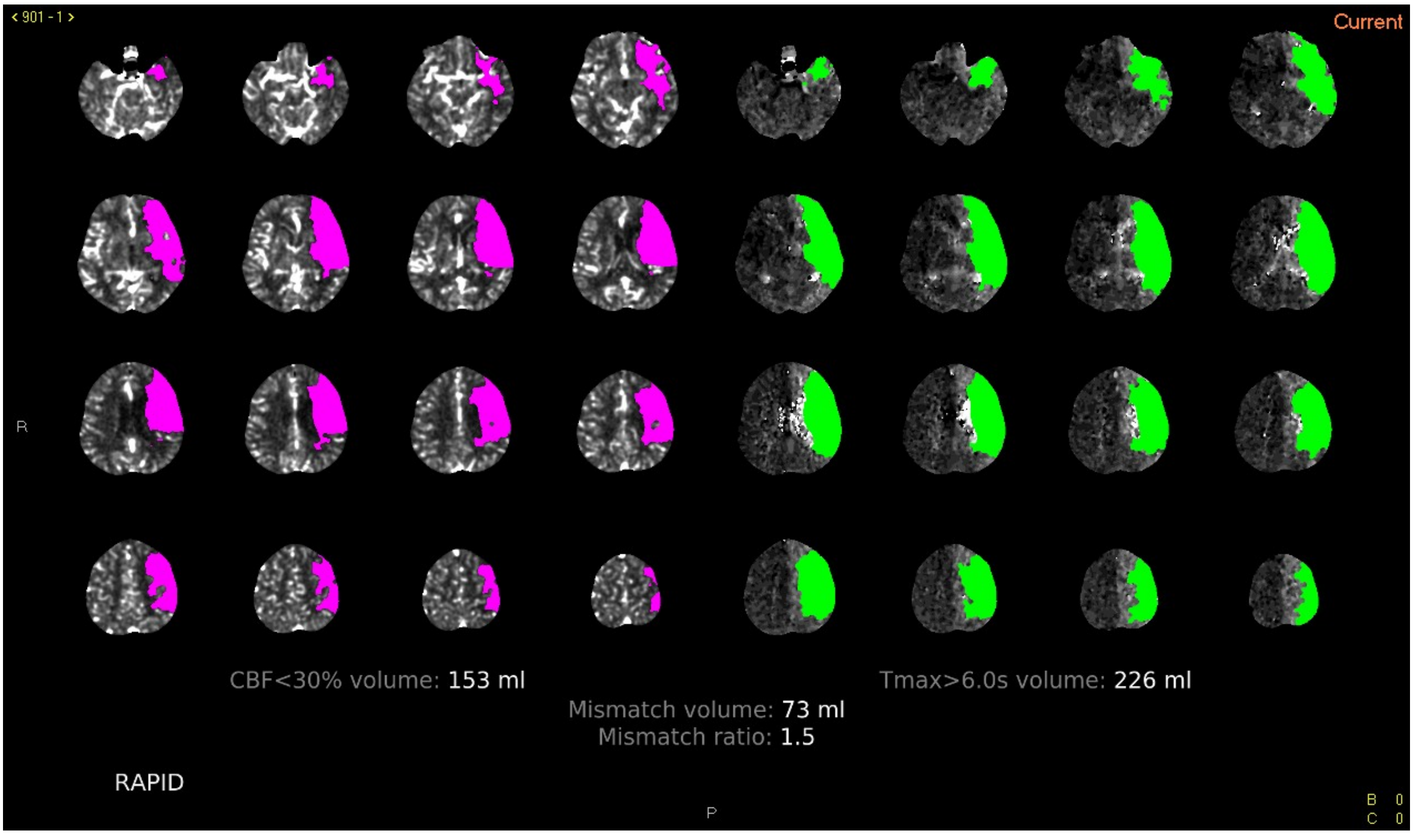

Based on the clinical and radiologic details will you recommend endovascular thrombectomy for this patient?

- ◯ Yes
- ◯ No

Case 9: 40

Case Details
Demographics 88-year-old female
Occlusion Left carotid termination
NIHSS 25
Last known well time 07:30
Time of CT scout film (Start of CT) 20:33

Non-contrast CT

What is the ASPECTS of this scan?

____

Single Phase CTA

What is the single-phase collateral score for this scan?

- ◯ Collateral Score 0: Absence of vessels on CTA distal to the occlusion
- ◯ Collateral Score 1: Collateral supply filling < or equal to 50% but >0% of the occluded MCA territory.
- ◯ Collateral Score 2: Collateral supply filling >50% but < 100% of the occluded MCA territory
- ◯ Collateral Score 3: 100% collateral supply of the occluded MCA territory

---

Based on the clinical and radiologic details will you recommend endovascular thrombectomy for this patient?

- ◯ Yes
- ◯ No

Case 10: 05

Case Details
Demographics 41-year-old male
Occlusion Right carotid terminus
NIHSS 08
Last known well time 08:00
Time of CT scout film (Start of CT) 17:42

Non-contrast CT

What is the ASPECTS of this scan? ______________________________

Single Phase CTA

What is the single-phase collateral score for this scan?

- ◯ Collateral Score 0: Absence of vessels on CTA distal to the occlusion
- ◯ Collateral Score 1: Collateral supply filling < or equal to 50% but >0% of the occluded MCA territory.
- ◯ Collateral Score 2: Collateral supply filling >50% but < 100% of the occluded MCA territory
- ◯ Collateral Score 3: 100% collateral supply of the occluded MCA territory

Automate Perfusion Scan Results

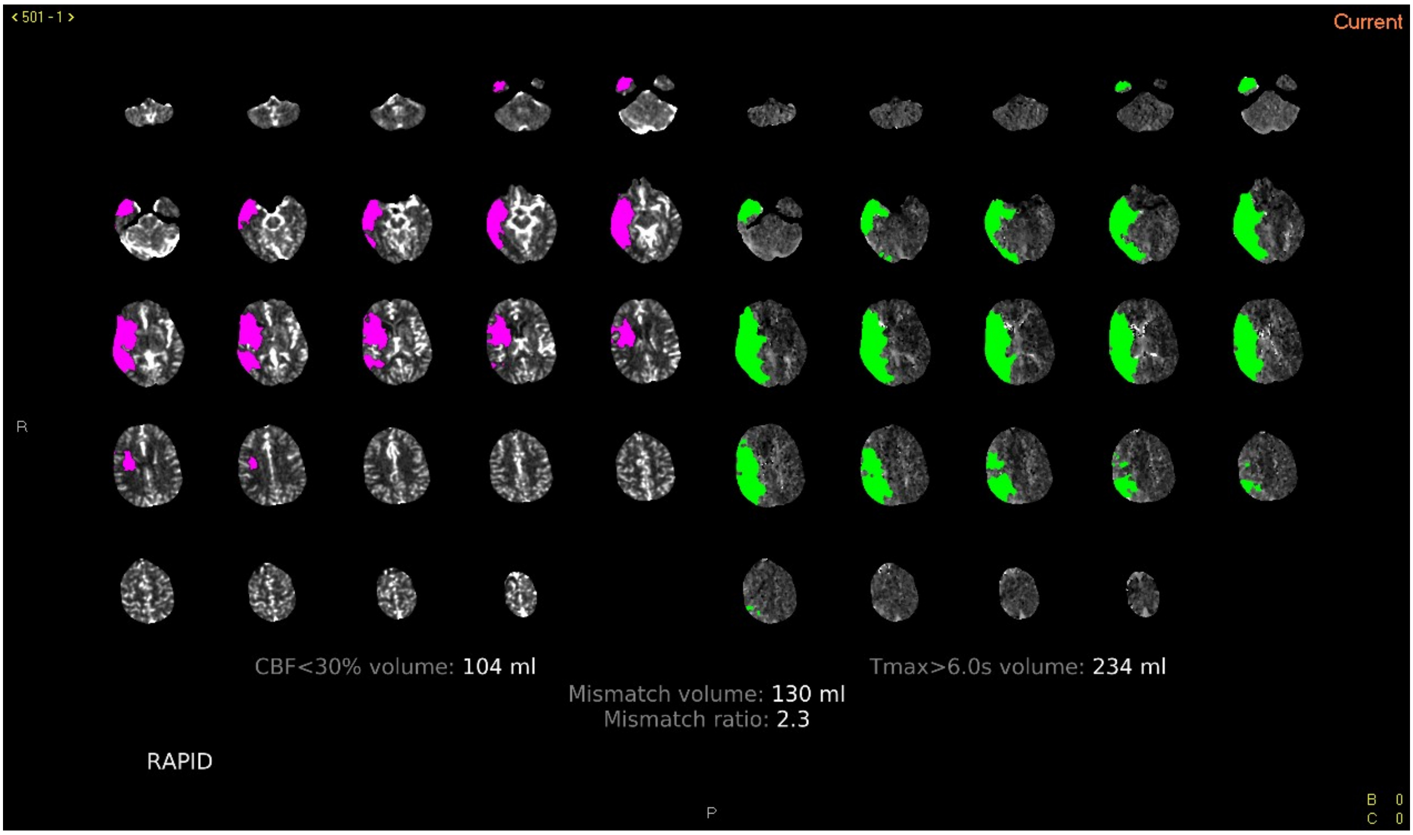

Based on the clinical and radiologic details will you recommend endovascular thrombectomy for this patient?

- ◯ Yes
- ◯ No

Case 11: 24

Case Details
Demographics 84-year-old female
Occlusion Left tandem
NIHSS 26
Last known well time 18:00 of the night before
Time of CT scout film (Start of CT) 15:30 of the current day

Non-contrast CT

What is the ASPECTS of this scan? __________________________________

Single Phase CTA

What is the single-phase collateral score for this scan?

- ◯ Collateral Score 0: Absence of vessels on CTA distal to the occlusion
- ◯ Collateral Score 1: Collateral supply filling < or equal to 50% but >0% of the occluded MCA territory.
- ◯ Collateral Score 2: Collateral supply filling >50% but < 100% of the occluded MCA territory
- ◯ Collateral Score 3: 100% collateral supply of the occluded MCA territory

Based on the clinical and radiologic details will you recommend endovascular thrombectomy for this patient?

- ◯ Yes
- ◯ No

Case 12: 01

Case Details
Demographics 87-year-old female
Occlusion Left carotid termination
NIHSS 27
Last known well time 21:10 The night prior
Time of CT scout film (Start of CT) 04:07 of the current day

Non-contrast CT

What is the ASPECTS of this scan?

____

Single Phase CTA

What is the single-phase collateral score for this scan?

- ◯ Collateral Score 0: Absence of vessels on CTA distal to the occlusion
- ◯ Collateral Score 1: Collateral supply filling < or equal to 50% but >0% of the occluded MCA territory.
- ◯ Collateral Score 2: Collateral supply filling >50% but < 100% of the occluded MCA territory
- ◯ Collateral Score 3: 100% collateral supply of the occluded MCA territory

Automate Perfusion Scan Results

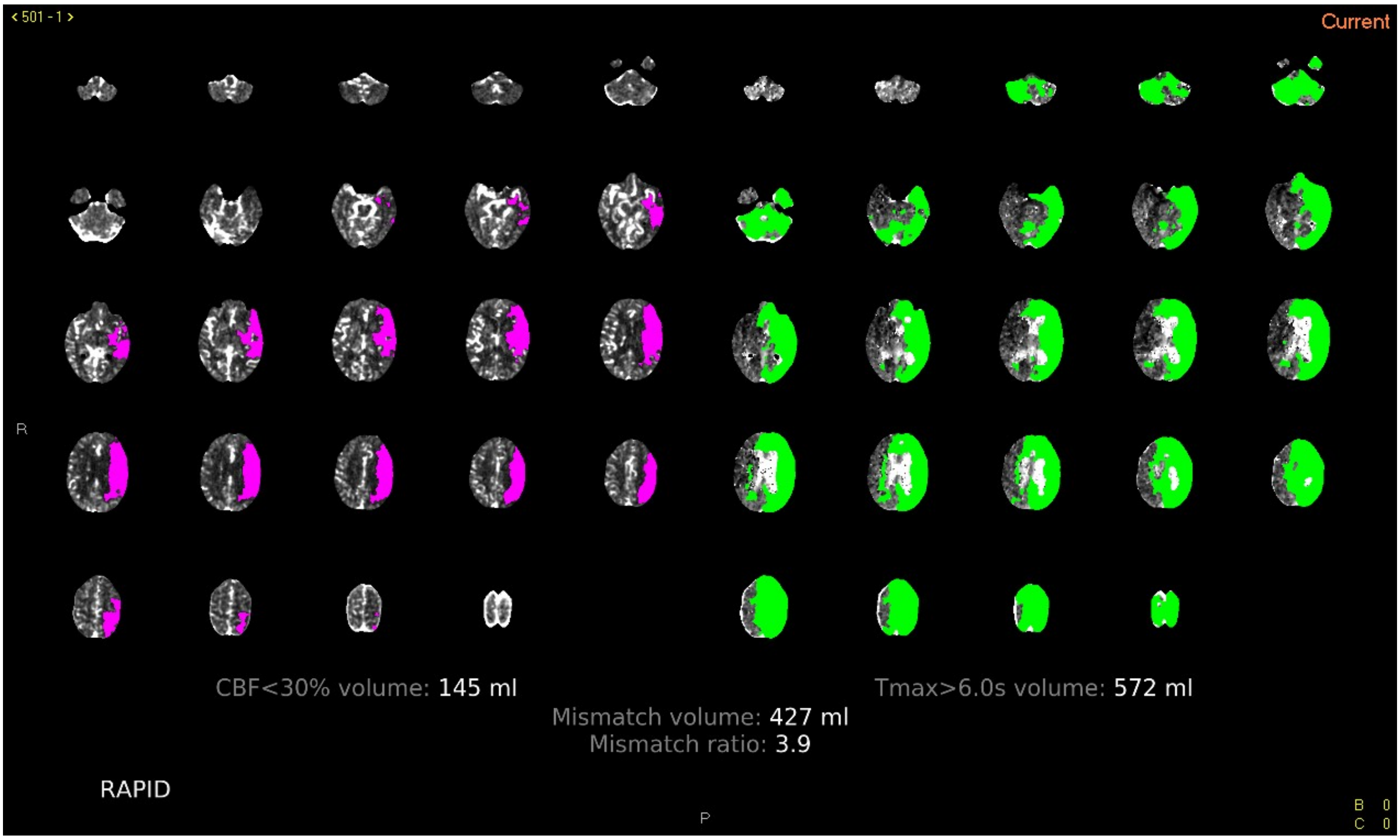

Based on the clinical and radiologic details will you recommend endovascular thrombectomy for this patient?

- ◯ Yes
- ◯ No

Case 13: 50

Case Details
Demographics 89-year-old male
Occlusion Right M1
NIHSS 18
Last known well time 23:00 The night prior
Time of CT scout film (Start of CT) 15:27 of the current day

Non-contrast CT

What is the ASPECTS of this scan? ________________________

Single Phase CTA

What is the single-phase collateral score for this scan?

- ◯ Collateral Score 0: Absence of vessels on CTA distal to the occlusion
- ◯ Collateral Score 1: Collateral supply filling < or equal to 50% but >0% of the occluded MCA territory.
- ◯ Collateral Score 2: Collateral supply filling >50% but < 100% of the occluded MCA territory
- ◯ Collateral Score 3: 100% collateral supply of the occluded MCA territory

Based on the clinical and radiologic details will you recommend endovascular thrombectomy for this patient?

- ◯ Yes
- ◯ No

Case 14: 06

Case Details
Demographics 41-year-old male
Occlusion Right carotid termination
NIHSS 8
Last known well time 08:00
Time of CT scout film (Start of CT) 17:42

Non-contrast CT

What is the ASPECTS of this scan?

____

Single Phase CTA

What is the single-phase collateral score for this scan?

- ◯ Collateral Score 0: Absence of vessels on CTA distal to the occlusion
- ◯ Collateral Score 1: Collateral supply filling < or equal to 50% but >0% of the occluded MCA territory.
- ◯ Collateral Score 2: Collateral supply filling >50% but < 100% of the occluded MCA territory
- ◯ Collateral Score 3: 100% collateral supply of the occluded MCA territory

Based on the clinical and radiologic details will you recommend endovascular thrombectomy for this patient?

- ◯ Yes
- ◯ No

Case 15: 27

Case Details
Demographics 40-year-old female
Occlusion Left M1
NIHSS 8
Last known well time 23:30 The night prior
Time of CT scout film (Start of CT) 09:44 of the current day

Non-contrast CT

What is the ASPECTS of this scan? __________________________________

Single Phase CTA

What is the single-phase collateral score for this scan?

- ◯ Collateral Score 0: Absence of vessels on CTA distal to the occlusion
- ◯ Collateral Score 1: Collateral supply filling < or equal to 50% but >0% of the occluded MCA territory.
- ◯ Collateral Score 2: Collateral supply filling >50% but < 100% of the occluded MCA territory
- ◯ Collateral Score 3: 100% collateral supply of the occluded MCA territory

Automate Perfusion Scan Results

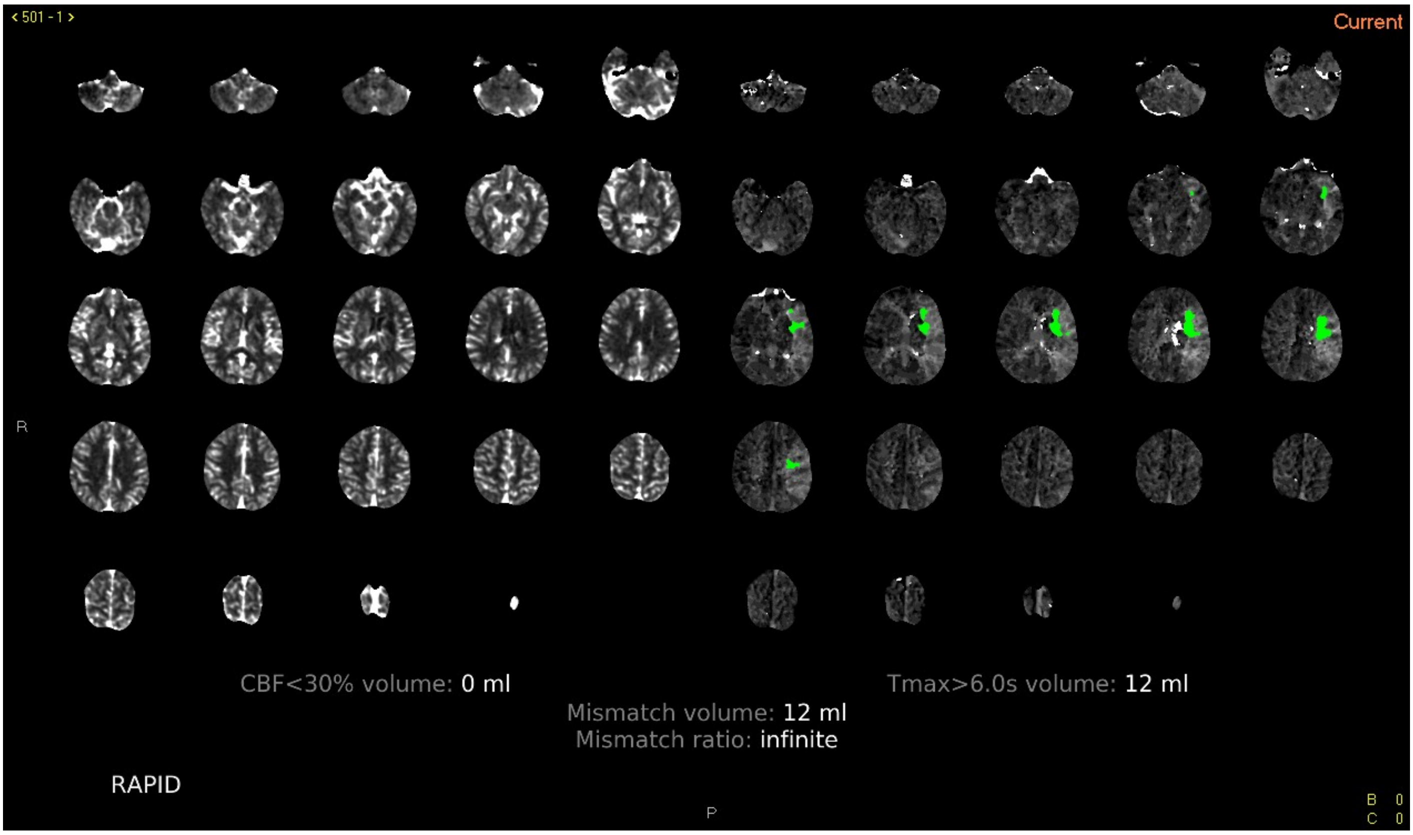

Based on the clinical and radiologic details will you recommend endovascular thrombectomy for this patient?

- ◯ Yes
- ◯ No

Case 16: 20

Case Details
Demographics 57-year-old female
Occlusion Left carotid termination
NIHSS 13
Last known well time 02:30
Time of CT scout film (Start of CT) 23:21

Non-contrast CT

What is the ASPECTS of this scan? ____________________

Single Phase CTA

What is the single-phase collateral score for this scan?

- ◯ Collateral Score 0: Absence of vessels on CTA distal to the occlusion
- ◯ Collateral Score 1: Collateral supply filling < or equal to 50% but >0% of the occluded MCA territory.
- ◯ Collateral Score 2: Collateral supply filling >50% but < 100% of the occluded MCA territory
- ◯ Collateral Score 3: 100% collateral supply of the occluded MCA territory

---

Based on the clinical and radiologic details will you recommend endovascular thrombectomy for this patient?

- ◯ Yes
- ◯ No

Case 17: 18

Case Details
Demographics 76-year-old female
Occlusion Left M1
NIHSS 25
Last known well time 23:50 The night prior
Time of CT scout film (Start of CT) 16:40 of the current day

Non-contrast CT

What is the ASPECTS of this scan? __________________________________

Single Phase CTA

What is the single-phase collateral score for this scan?

- ◯ Collateral Score 0: Absence of vessels on CTA distal to the occlusion
- ◯ Collateral Score 1: Collateral supply filling < or equal to 50% but >0% of the occluded MCA territory.
- ◯ Collateral Score 2: Collateral supply filling >50% but < 100% of the occluded MCA territory
- ◯ Collateral Score 3: 100% collateral supply of the occluded MCA territory

Based on the clinical and radiologic details will you recommend endovascular thrombectomy for this patient?

- ◯ Yes
- ◯ No

Case 18: 49

Case Details
Demographics 89-year-old male
Occlusion Right M1
NIHSS 18
Last known well time 23:00 The night prior
Time of CT scout film (Start of CT) 15:27 of the current day

Non-contrast CT

What is the ASPECTS of this scan? __________________________________

Single Phase CTA

What is the single-phase collateral score for this scan?

- ◯ Collateral Score 0: Absence of vessels on CTA distal to the occlusion
- ◯ Collateral Score 1: Collateral supply filling < or equal to 50% but >0% of the occluded MCA territory.
- ◯ Collateral Score 2: Collateral supply filling >50% but < 100% of the occluded MCA territory
- ◯ Collateral Score 3: 100% collateral supply of the occluded MCA territory

Automate Perfusion Scan Results

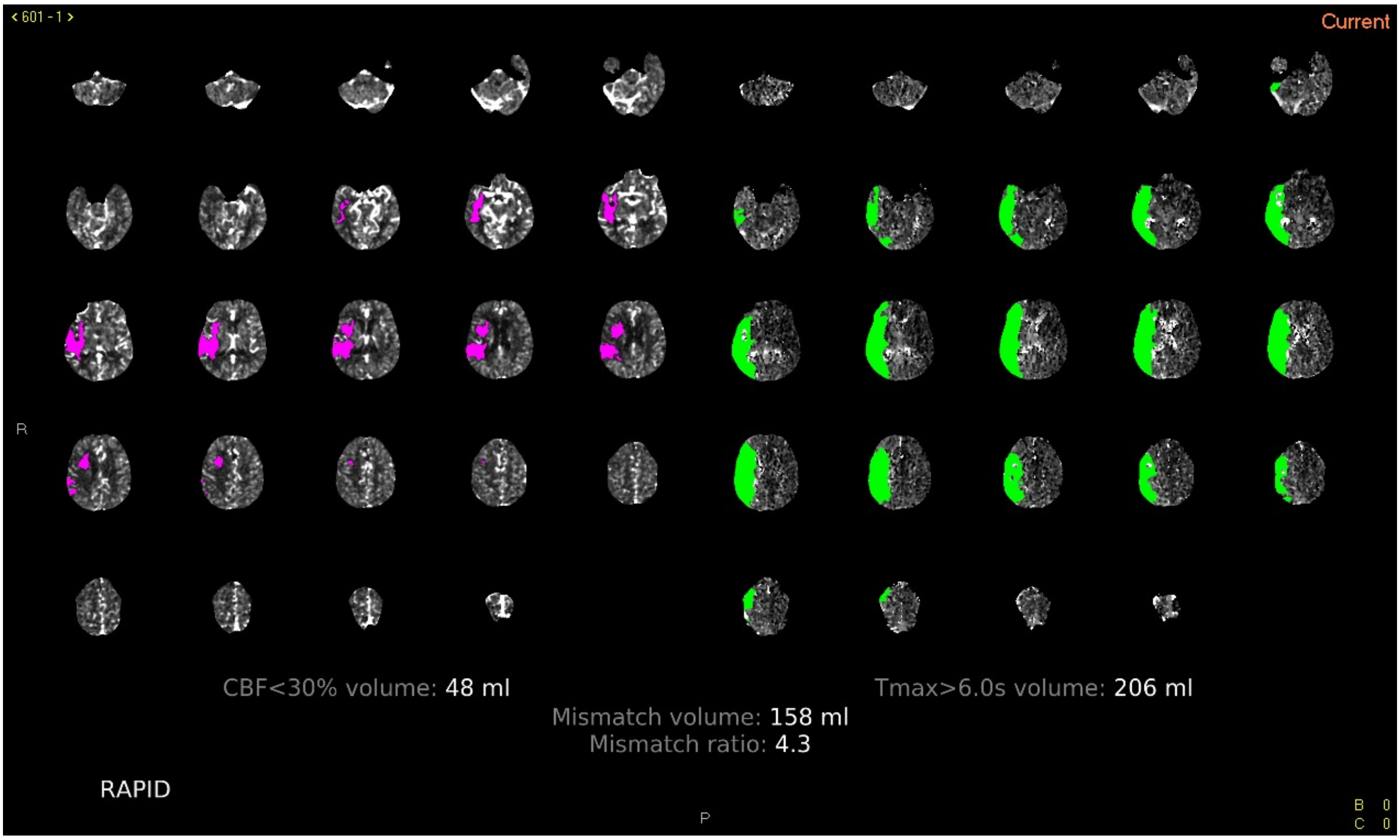

Based on the clinical and radiologic details will you recommend endovascular thrombectomy for this patient?

- ◯ Yes
- ◯ No

Case 19: 19

Case Details
Demographics 57-year-old female
Occlusion Left carotid termination
NIHSS 13
Last known well time 02:30 The night prior
Time of CT scout film (Start of CT) 23:21 of the current day

Non-contrast CT

What is the ASPECTS of this scan? ________________________

Single Phase CTA

What is the single-phase collateral score for this scan?

- ◯ Collateral Score 0: Absence of vessels on CTA distal to the occlusion
- ◯ Collateral Score 1: Collateral supply filling < or equal to 50% but >0% of the occluded MCA territory.
- ◯ Collateral Score 2: Collateral supply filling >50% but < 100% of the occluded MCA territory
- ◯ Collateral Score 3: 100% collateral supply of the occluded MCA territory

Automate Perfusion Scan Results

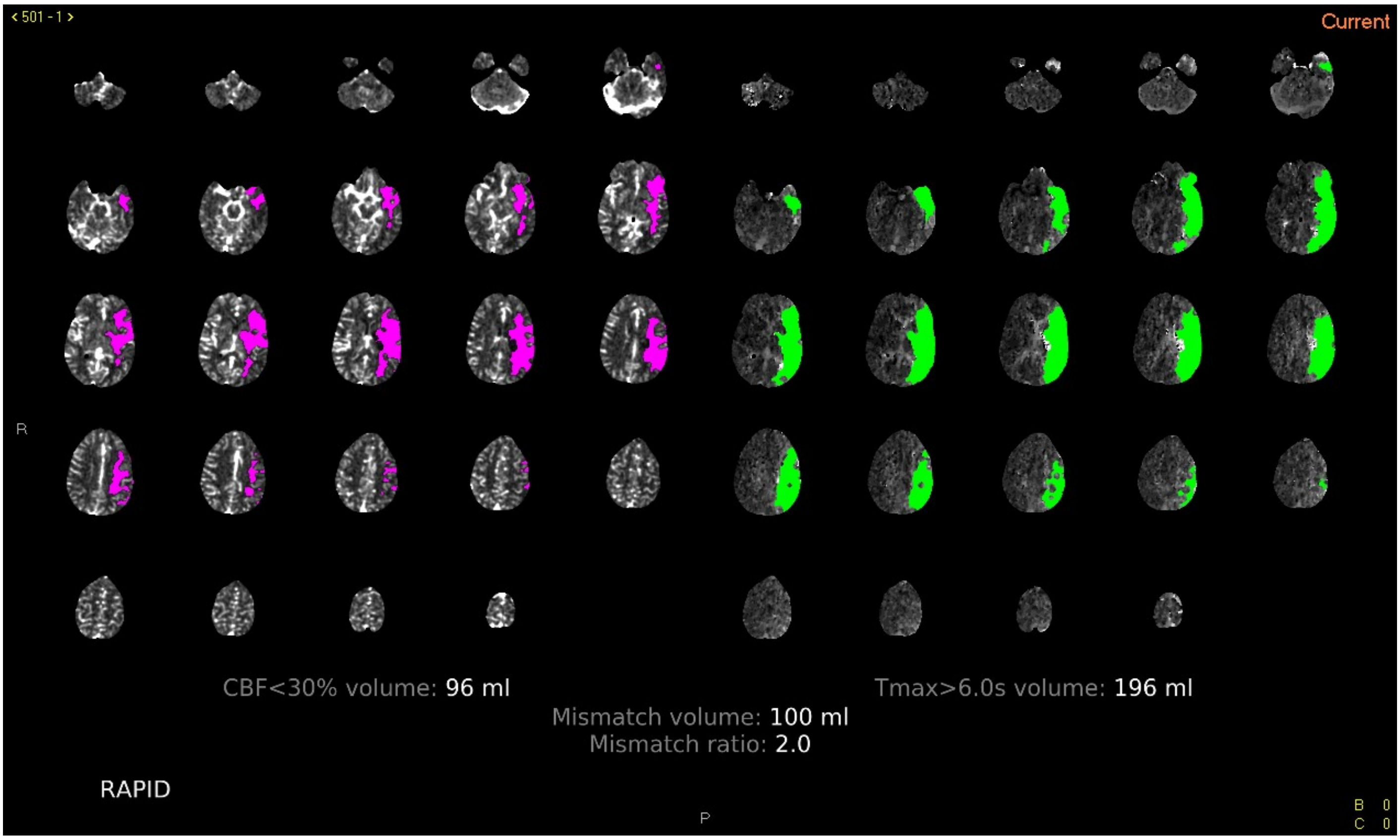

Based on the clinical and radiologic details will you recommend endovascular thrombectomy for this patient?

- ◯ Yes
- ◯ No

Case 20: 11

Case Details
Demographics 82-year-old female
Occlusion Right carotid termination
NIHSS 22
Last known well time 08:00
Time of CT scout film (Start of CT) 21:23 of the current day

Non-contrast CT

What is the ASPECTS of this scan?

____

Single Phase CTA

What is the single-phase collateral score for this scan?

- ◯ Collateral Score 0: Absence of vessels on CTA distal to the occlusion
- ◯ Collateral Score 1: Collateral supply filling < or equal to 50% but >0% of the occluded MCA territory.
- ◯ Collateral Score 2: Collateral supply filling >50% but < 100% of the occluded MCA territory
- ◯ Collateral Score 3: 100% collateral supply of the occluded MCA territory

Automate Perfusion Scan Results

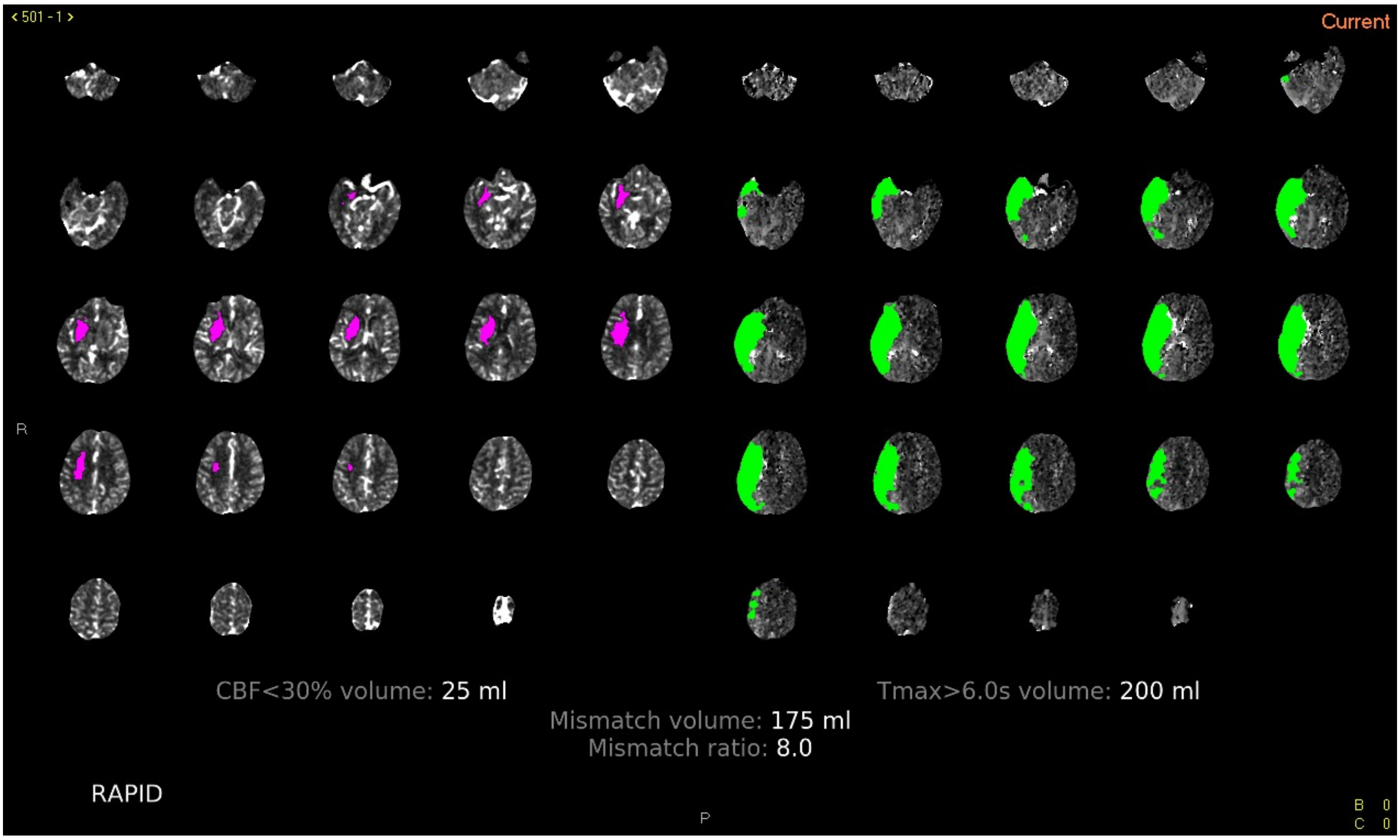

Based on the clinical and radiologic details will you recommend endovascular thrombectomy for this patient?

- ◯ Yes
- ◯ No

Case 21: 52

Case Details
Demographics 51-year-old male
Occlusion Right M1
NIHSS 13
Last known well time 23:00 The night prior
Time of CT scout film (Start of CT) 16:25 of the current day

Non-contrast CT

What is the ASPECTS of this scan? __________________________________

Single Phase CTA

What is the single-phase collateral score for this scan?

- ◯ Collateral Score 0: Absence of vessels on CTA distal to the occlusion
- ◯ Collateral Score 1: Collateral supply filling < or equal to 50% but >0% of the occluded MCA territory.
- ◯ Collateral Score 2: Collateral supply filling >50% but < 100% of the occluded MCA territory
- ◯ Collateral Score 3: 100% collateral supply of the occluded MCA territory

Based on the clinical and radiologic details will you recommend endovascular thrombectomy for this patient?

- ◯ Yes
- ◯ No

Case 22: 10

Case Details
Demographics 39-year-old female
Occlusion Left M1
NIHSS 9
Last known well time 22:00 The night prior
Time of CT scout film (Start of CT) 13:12 of the current day

Non-contrast CT

What is the ASPECTS of this scan? __________________________________

Single Phase CTA

What is the single-phase collateral score for this scan?

- ◯ Collateral Score 0: Absence of vessels on CTA distal to the occlusion
- ◯ Collateral Score 1: Collateral supply filling < or equal to 50% but >0% of the occluded MCA territory.
- ◯ Collateral Score 2: Collateral supply filling >50% but < 100% of the occluded MCA territory
- ◯ Collateral Score 3: 100% collateral supply of the occluded MCA territory

Based on the clinical and radiologic details will you recommend endovascular thrombectomy for this patient?

- ◯ Yes
- ◯ No

Case 23: 37

Case Details
Demographics 75-year-old male
Occlusion Right tandem occlusion
NIHSS 24
Last known well time 18:00 The night prior
Time of CT scout film (Start of CT) 13:00 of the current day

Non-contrast CT

What is the ASPECTS of this scan? ______________________

Single Phase CTA

What is the single-phase collateral score for this scan?

- ◯ Collateral Score 0: Absence of vessels on CTA distal to the occlusion
- ◯ Collateral Score 1: Collateral supply filling < or equal to 50% but >0% of the occluded MCA territory.
- ◯ Collateral Score 2: Collateral supply filling >50% but < 100% of the occluded MCA territory
- ◯ Collateral Score 3: 100% collateral supply of the occluded MCA territory

Automate Perfusion Scan Results

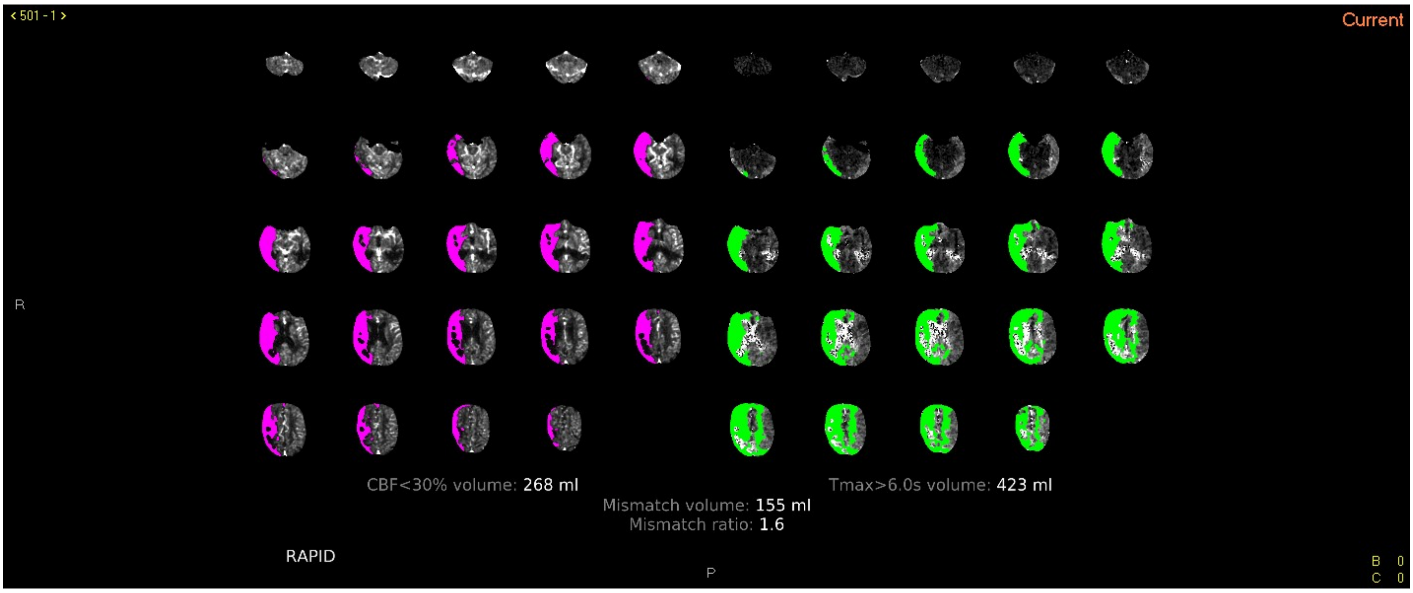

Based on the clinical and radiologic details will you recommend endovascular thrombectomy for this patient?

- ◯ Yes
- ◯ No

Case 24: 54

Case Details
Demographics 81-year-old female
Occlusion Left carotid termination
NIHSS 18
Last known well time 21:30 The night prior
Time of CT scout film (Start of CT) 05:58 of the current day

Non-contrast CT

What is the ASPECTS of this scan? ______________________

Single Phase CTA

What is the single-phase collateral score for this scan?

- ◯ Collateral Score 0: Absence of vessels on CTA distal to the occlusion
- ◯ Collateral Score 1: Collateral supply filling < or equal to 50% but >0% of the occluded MCA territory.
- ◯ Collateral Score 2: Collateral supply filling >50% but < 100% of the occluded MCA territory
- ◯ Collateral Score 3: 100% collateral supply of the occluded MCA territory

Based on the clinical and radiologic details will you recommend endovascular thrombectomy for this patient?

- ◯ Yes
- ◯ No

Case 25: 22

Case Details
Demographics 84-year-old male
Occlusion Left M1
NIHSS 11
Last known well time 02:00
Time of CT scout film (Start of CT) 17:29

Non-contrast CT

What is the ASPECTS of this scan? __________________________________

Single Phase CTA

What is the single-phase collateral score for this scan?

- ◯ Collateral Score 0: Absence of vessels on CTA distal to the occlusion
- ◯ Collateral Score 1: Collateral supply filling < or equal to 50% but >0% of the occluded MCA territory.
- ◯ Collateral Score 2: Collateral supply filling >50% but < 100% of the occluded MCA territory
- ◯ Collateral Score 3: 100% collateral supply of the occluded MCA territory

Based on the clinical and radiologic details will you recommend endovascular thrombectomy for this patient?

- ◯ Yes
- ◯ No

Case 26: 47

Case Details
Demographics 82-year-old male
Occlusion Left M1
NIHSS 14
Last known well time 08:00
Time of CT scout film (Start of CT) 22:02

Non-contrast CT

What is the ASPECTS of this scan? __________________________________

Single Phase CTA

What is the single-phase collateral score for this scan?

- ◯ Collateral Score 0: Absence of vessels on CTA distal to the occlusion
- ◯ Collateral Score 1: Collateral supply filling < or equal to 50% but >0% of the occluded MCA territory.
- ◯ Collateral Score 2: Collateral supply filling >50% but < 100% of the occluded MCA territory
- ◯ Collateral Score 3: 100% collateral supply of the occluded MCA territory

Automate Perfusion Scan Results

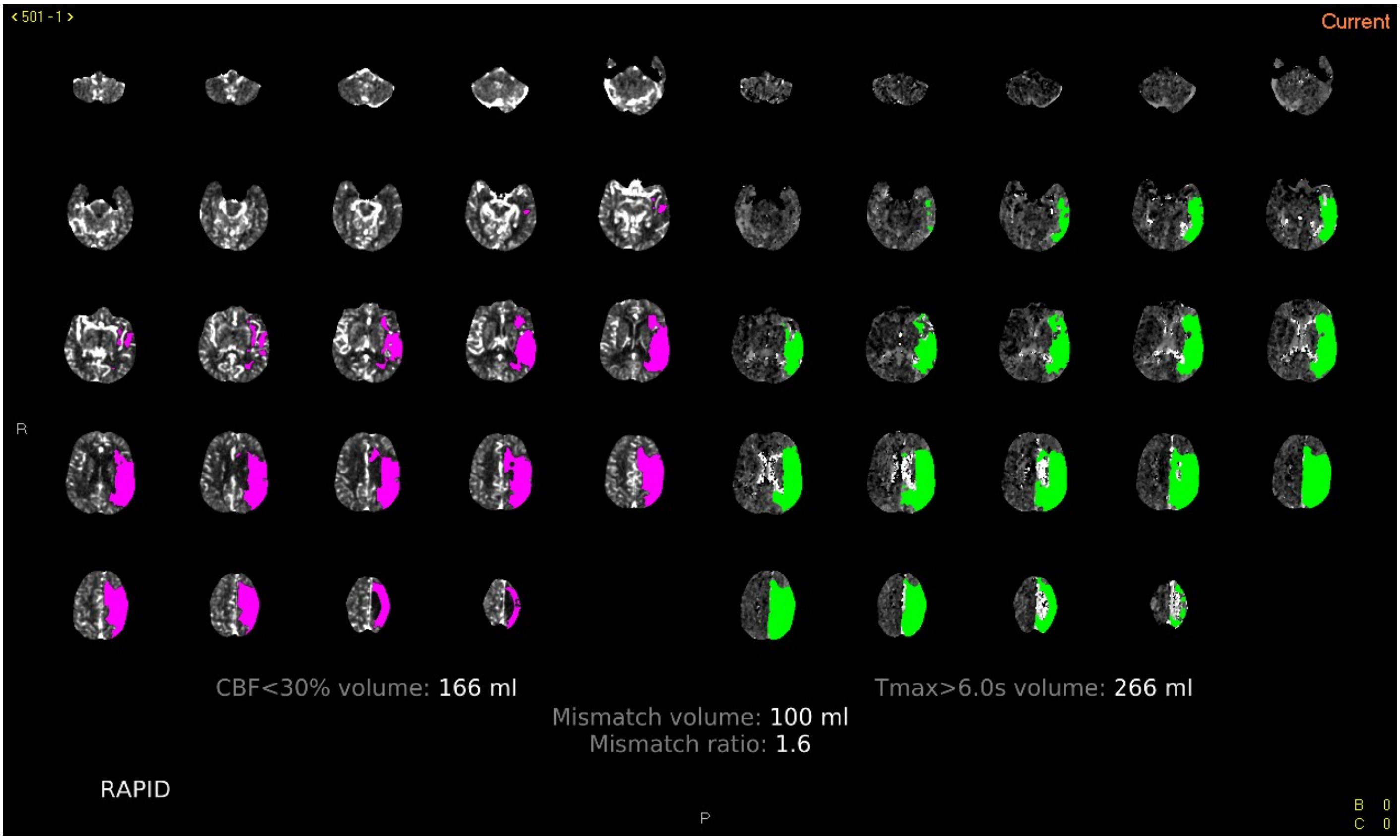

Based on the clinical and radiologic details will you recommend endovascular thrombectomy for this patient?

- ◯ Yes
- ◯ No

Case 27: 15

Case Details
Demographics 83-year-old male
Occlusion Right M1
NIHSS 15
Last known well time 13:30
Time of CT scout film (Start of CT) 20:39

Non-contrast CT

What is the ASPECTS of this scan?

____

Single Phase CTA

What is the single-phase collateral score for this scan?

- ◯ Collateral Score 0: Absence of vessels on CTA distal to the occlusion
- ◯ Collateral Score 1: Collateral supply filling < or equal to 50% but >0% of the occluded MCA territory.
- ◯ Collateral Score 2: Collateral supply filling >50% but < 100% of the occluded MCA territory
- ◯ Collateral Score 3: 100% collateral supply of the occluded MCA territory

Automate Perfusion Scan Results

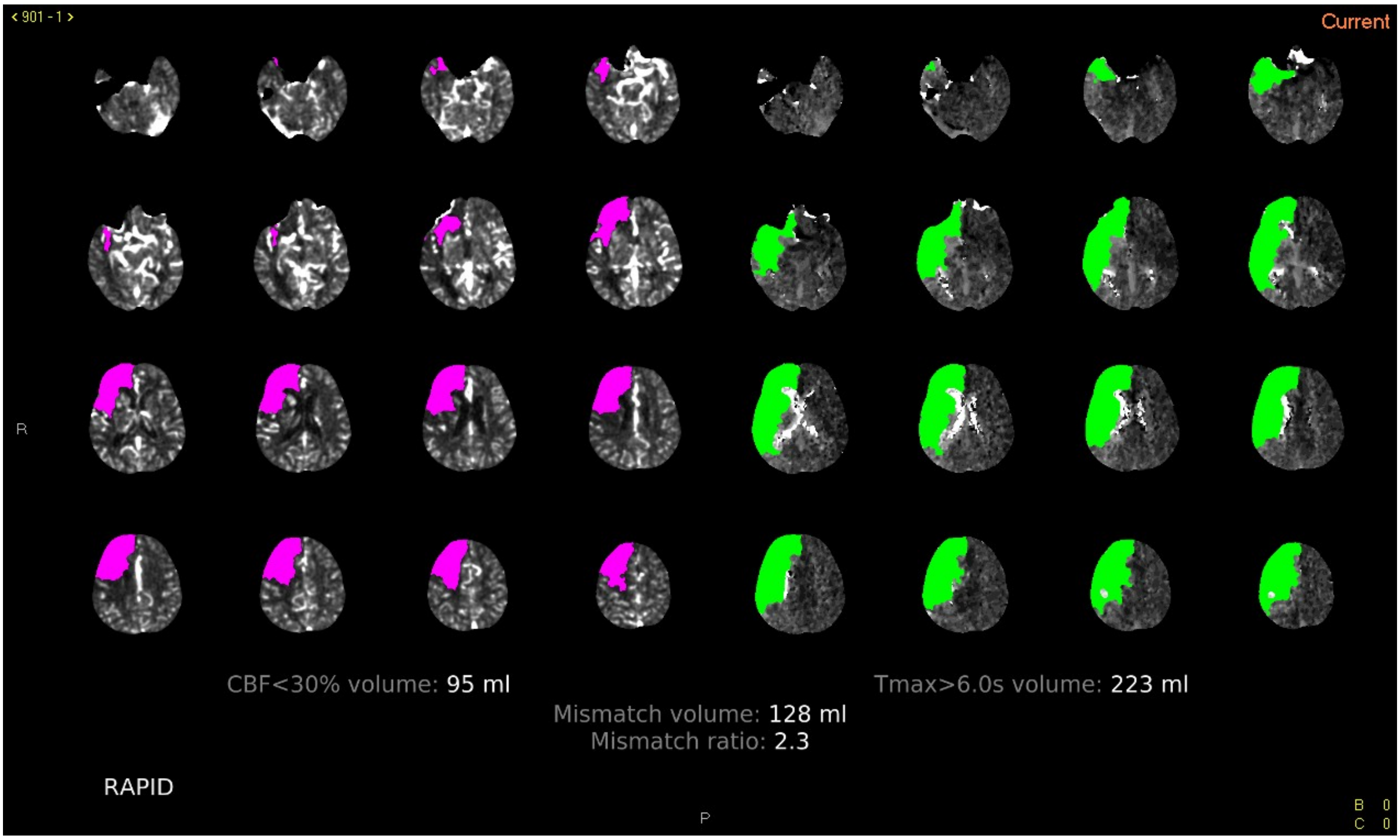

Based on the clinical and radiologic details will you recommend endovascular thrombectomy for this patient?

- ◯ Yes
- ◯ No

Case 28: 04

Case Details
Demographics 82-year-old male
Occlusion Right M1
NIHSS 14
Last known well time 00:10
Time of CT scout film (Start of CT) 15:13

Non-contrast CT

What is the ASPECTS of this scan? __________________________________

Single Phase CTA

What is the single-phase collateral score for this scan?

- ◯ Collateral Score 0: Absence of vessels on CTA distal to the occlusion
- ◯ Collateral Score 1: Collateral supply filling < or equal to 50% but >0% of the occluded MCA territory.
- ◯ Collateral Score 2: Collateral supply filling >50% but < 100% of the occluded MCA territory
- ◯ Collateral Score 3: 100% collateral supply of the occluded MCA territory

Based on the clinical and radiologic details will you recommend endovascular thrombectomy for this patient?

- ◯ Yes
- ◯ No

Case 29: 17

Case Details
Demographics 76-year-old female
Occlusion Left M1
NIHSS 25
Last known well time 23:50
Time of CT scout film (Start of CT) 16:40

Non-contrast CT

What is the ASPECTS of this scan?

____

Single Phase CTA

What is the single-phase collateral score for this scan?

- ◯ Collateral Score 0: Absence of vessels on CTA distal to the occlusion
- ◯ Collateral Score 1: Collateral supply filling < or equal to 50% but >0% of the occluded MCA territory.
- ◯ Collateral Score 2: Collateral supply filling >50% but < 100% of the occluded MCA territory
- ◯ Collateral Score 3: 100% collateral supply of the occluded MCA territory

Automate Perfusion Scan Results

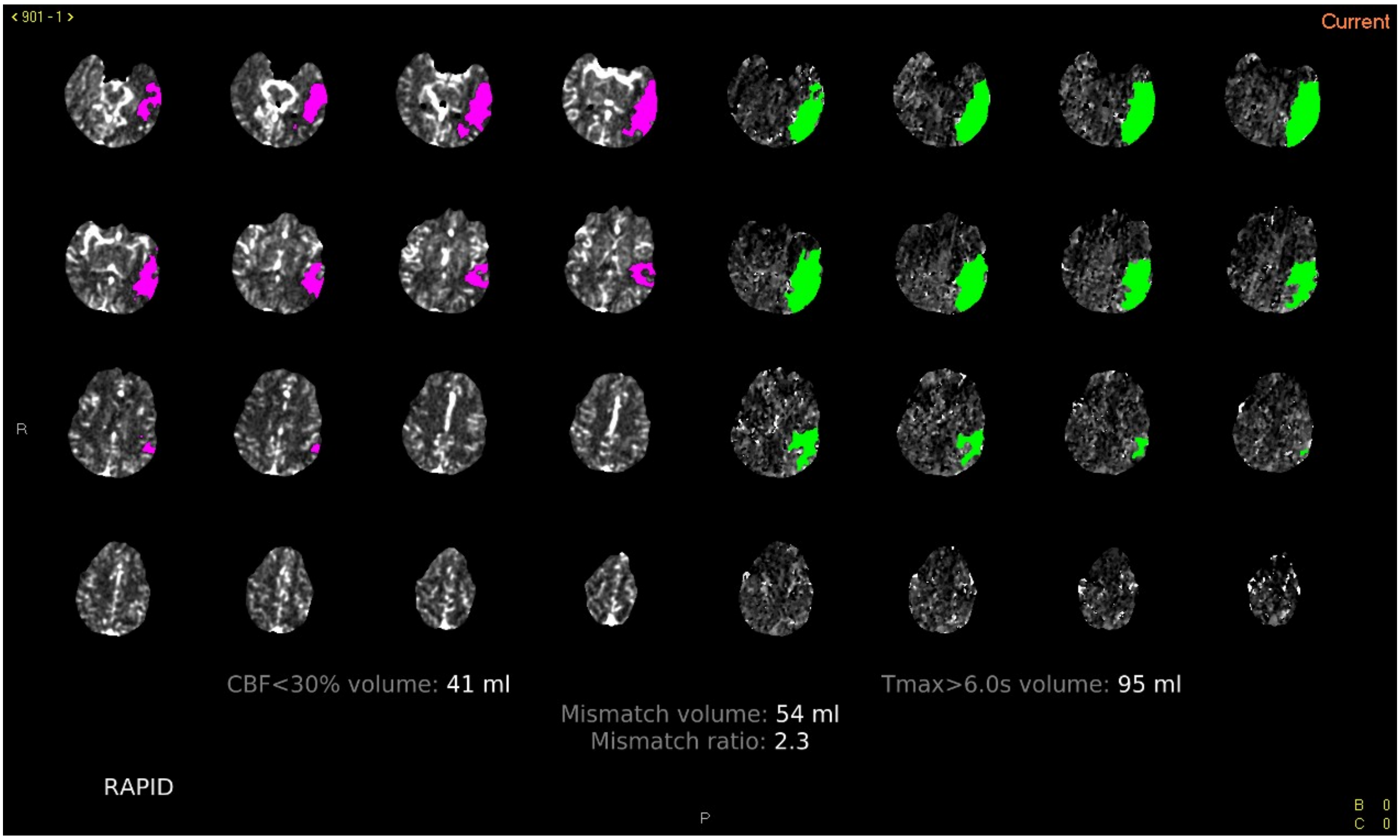

Based on the clinical and radiologic details will you recommend endovascular thrombectomy for this patient?

- ◯ Yes
- ◯ No

Case 30: 51

Case Details
Demographics 51-year-old male
Occlusion Right M1
NIHSS 13
Last known well time 23:00 The night prior
Time of CT scout film (Start of CT) 16:25 of the current day

Non-contrast CT

What is the ASPECTS of this scan? ______________________

Single Phase CTA

What is the single-phase collateral score for this scan?

- ◯ Collateral Score 0: Absence of vessels on CTA distal to the occlusion
- ◯ Collateral Score 1: Collateral supply filling < or equal to 50% but >0% of the occluded MCA territory.
- ◯ Collateral Score 2: Collateral supply filling >50% but < 100% of the occluded MCA territory
- ◯ Collateral Score 3: 100% collateral supply of the occluded MCA territory

Automate Perfusion Scan Results

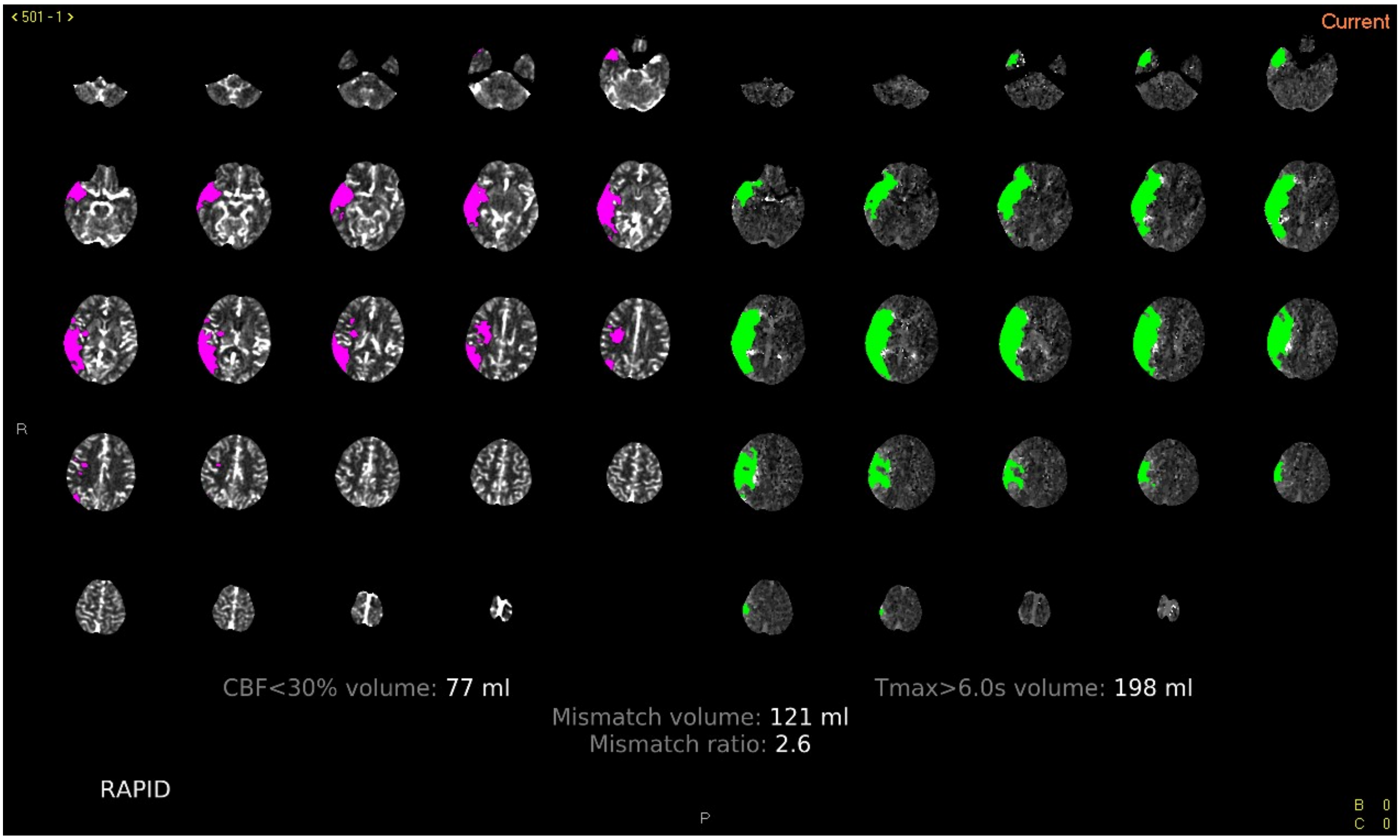

Based on the clinical and radiologic details will you recommend endovascular thrombectomy for this patient?

- ◯ Yes
- ◯ No

Case 31: 42

Case Details
Demographics 78-year-old female
Occlusion Right M1
NIHSS 15
Last known well time 15:15
Time of CT scout film (Start of CT) 20:19

Non-contrast CT

What is the ASPECTS of this scan? __________________________________

Single Phase CTA

What is the single-phase collateral score for this scan?

- ◯ Collateral Score 0: Absence of vessels on CTA distal to the occlusion
- ◯ Collateral Score 1: Collateral supply filling < or equal to 50% but >0% of the occluded MCA territory.
- ◯ Collateral Score 2: Collateral supply filling >50% but < 100% of the occluded MCA territory
- ◯ Collateral Score 3: 100% collateral supply of the occluded MCA territory

Based on the clinical and radiologic details will you recommend endovascular thrombectomy for this patient?

- ◯ Yes
- ◯ No

Case 32: 12

Case Details
Demographics 82-year-old female
Occlusion Right carotid termination
NIHSS 22
Last known well time 08:00
Time of CT scout film (Start of CT) 21:23

Non-contrast CT

What is the ASPECTS of this scan? ________________________

Single Phase CTA

What is the single-phase collateral score for this scan?

- ◯ Collateral Score 0: Absence of vessels on CTA distal to the occlusion
- ◯ Collateral Score 1: Collateral supply filling < or equal to 50% but >0% of the occluded MCA territory.
- ◯ Collateral Score 2: Collateral supply filling >50% but < 100% of the occluded MCA territory
- ◯ Collateral Score 3: 100% collateral supply of the occluded MCA territory

Based on the clinical and radiologic details will you recommend endovascular thrombectomy for this patient?

- ◯ Yes
- ◯ No

Case 33: 41

Case Details
Demographics 78-year-old female
Occlusion Right M1
NIHSS 15
Last known well time 15:15
Time of CT scout film (Start of CT) 20:19

Non-contrast CT

What is the ASPECTS of this scan? __________________________________

Single Phase CTA

What is the single-phase collateral score for this scan?

- ◯ Collateral Score 0: Absence of vessels on CTA distal to the occlusion
- ◯ Collateral Score 1: Collateral supply filling < or equal to 50% but >0% of the occluded MCA territory.
- ◯ Collateral Score 2: Collateral supply filling >50% but < 100% of the occluded MCA territory
- ◯ Collateral Score 3: 100% collateral supply of the occluded MCA territory

Automate Perfusion Scan Results

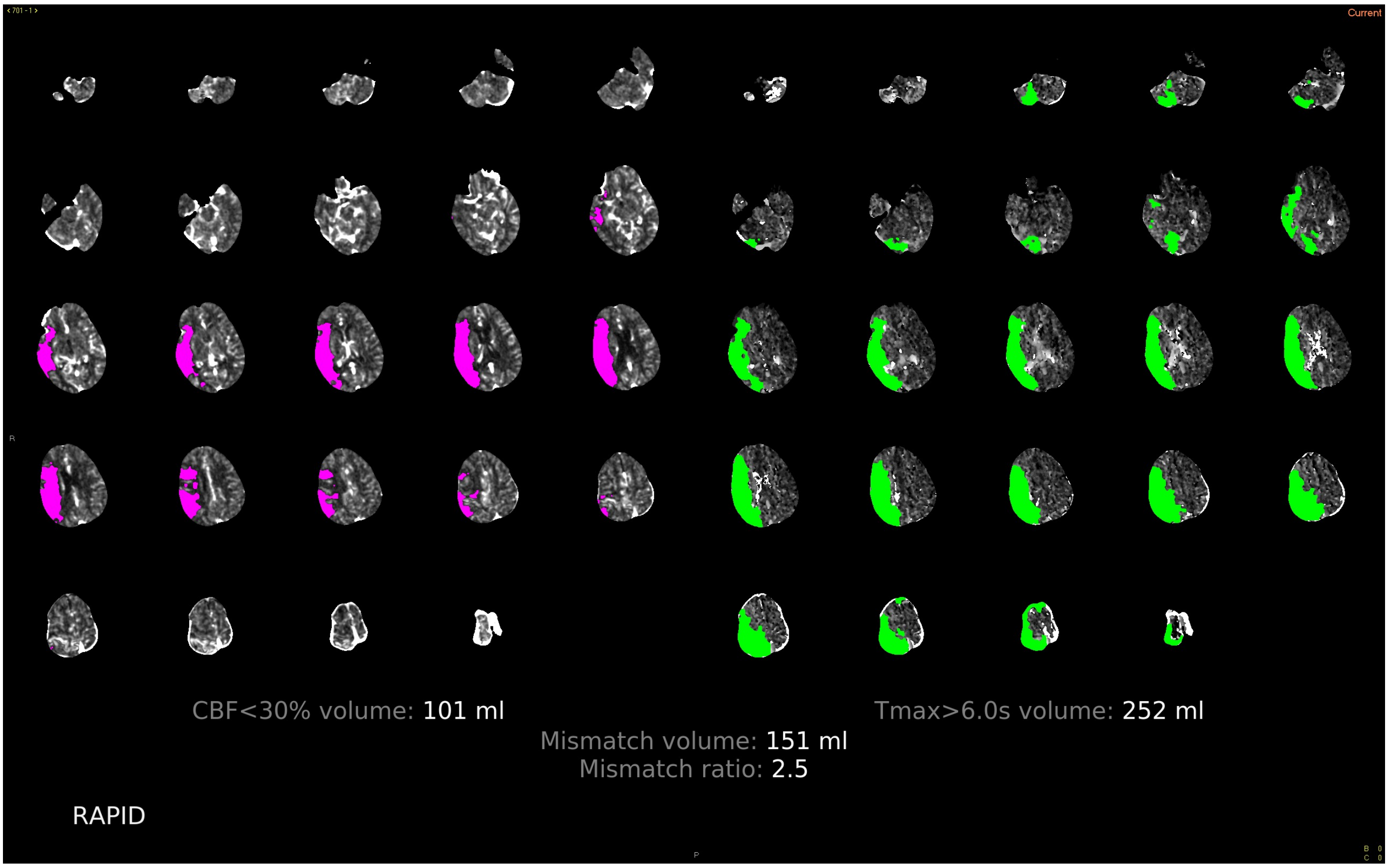

Based on the clinical and radiologic details will you recommend endovascular thrombectomy for this patient?

- ◯ Yes
- ◯ No

Case 34: 39

Case Details
Demographics 88-year-old female
Occlusion Left carotid termination
NIHSS 25
Last known well time 07:30
Time of CT scout film (Start of CT) 20:33

Non-contrast CT

What is the ASPECTS of this scan? ____________________

Single Phase CTA

What is the single-phase collateral score for this scan?

- ◯ Collateral Score 0: Absence of vessels on CTA distal to the occlusion
- ◯ Collateral Score 1: Collateral supply filling < or equal to 50% but >0% of the occluded MCA territory.
- ◯ Collateral Score 2: Collateral supply filling >50% but < 100% of the occluded MCA territory
- ◯ Collateral Score 3: 100% collateral supply of the occluded MCA territory

Automate Perfusion Scan Results

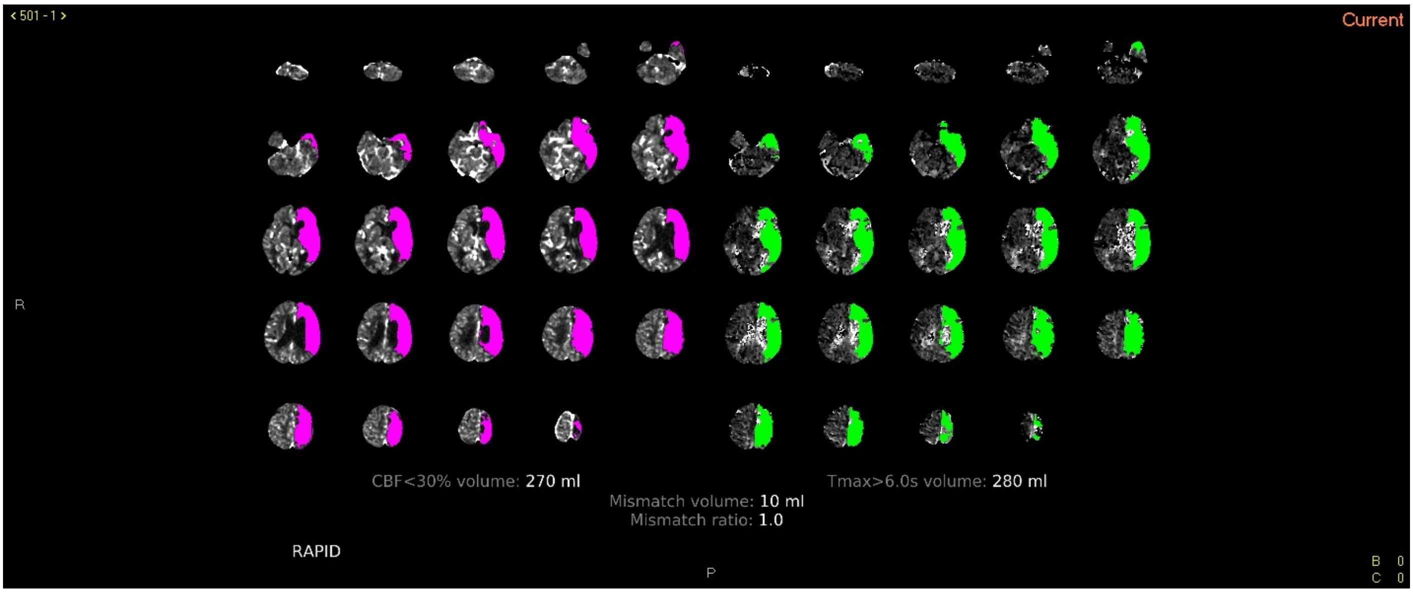

Based on the clinical and radiologic details will you recommend endovascular thrombectomy for this patient?

- ◯ Yes
- ◯ No

Case 35: 43

Case Details
Demographics 74-year-old male
Occlusion Right M1
NIHSS 20
Last known well time 19:00 the day prior
Time of CT scout film (Start of CT) 16:04 of the current day

Non-contrast CT

What is the ASPECTS of this scan? ______________________

Single Phase CTA

What is the single-phase collateral score for this scan?

- ◯ Collateral Score 0: Absence of vessels on CTA distal to the occlusion
- ◯ Collateral Score 1: Collateral supply filling < or equal to 50% but >0% of the occluded MCA territory.
- ◯ Collateral Score 2: Collateral supply filling >50% but < 100% of the occluded MCA territory
- ◯ Collateral Score 3: 100% collateral supply of the occluded MCA territory

Automate Perfusion Scan Results

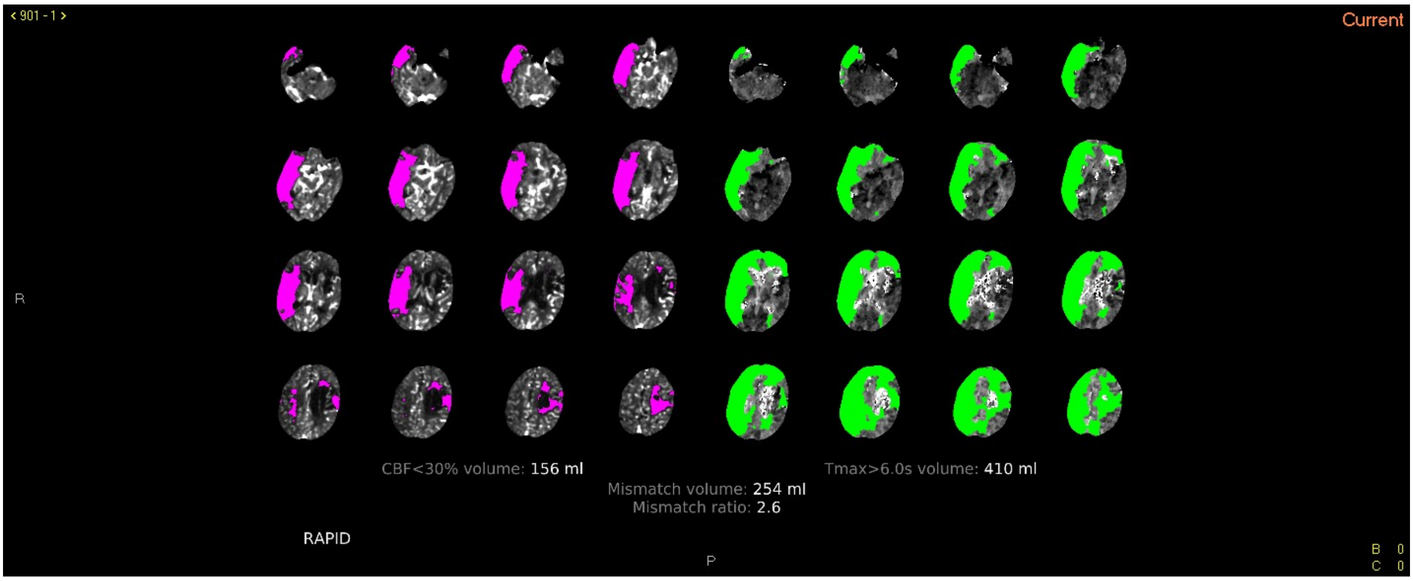

Based on the clinical and radiologic details will you recommend endovascular thrombectomy for this patient?

- ◯ Yes
- ◯ No

Case 36: 33

Case Details
Demographics 70-year-old male
Occlusion Left tandem occlusion
NIHSS 8
Last known well time 13:05 The day prior
Time of CT scout film (Start of CT) 00:36 of the current day

Non-contrast CT

What is the ASPECTS of this scan? ________________________________

Single Phase CTA

What is the single-phase collateral score for this scan?

- ◯ Collateral Score 0: Absence of vessels on CTA distal to the occlusion
- ◯ Collateral Score 1: Collateral supply filling < or equal to 50% but >0% of the occluded MCA territory.
- ◯ Collateral Score 2: Collateral supply filling >50% but < 100% of the occluded MCA territory
- ◯ Collateral Score 3: 100% collateral supply of the occluded MCA territory

Automate Perfusion Scan Results

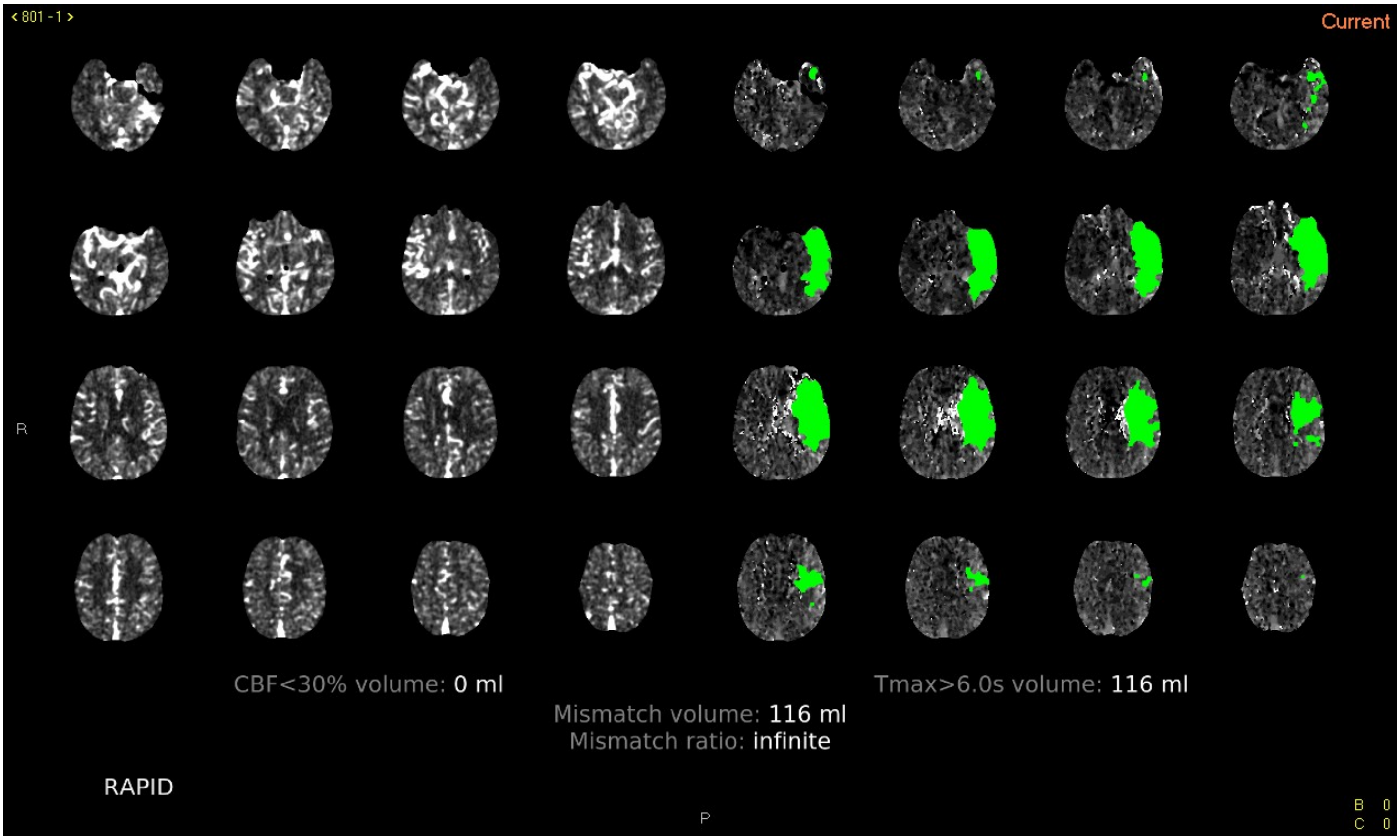

Based on the clinical and radiologic details will you recommend endovascular thrombectomy for this patient?

- ◯ Yes
- ◯ No

Case 37: 23

Case Details
Demographics 84-year-old female
Occlusion Left tandem
NIHSS 26
Last known well time 18:00 The night prior
Time of CT scout film (Start of CT) 15:30 of the current day

Non-contrast CT

What is the ASPECTS of this scan?

____

Single Phase CTA

What is the single-phase collateral score for this scan?

- ◯ Collateral Score 0: Absence of vessels on CTA distal to the occlusion
- ◯ Collateral Score 1: Collateral supply filling < or equal to 50% but >0% of the occluded MCA territory.
- ◯ Collateral Score 2: Collateral supply filling >50% but < 100% of the occluded MCA territory
- ◯ Collateral Score 3: 100% collateral supply of the occluded MCA territory

Automate Perfusion Scan Results

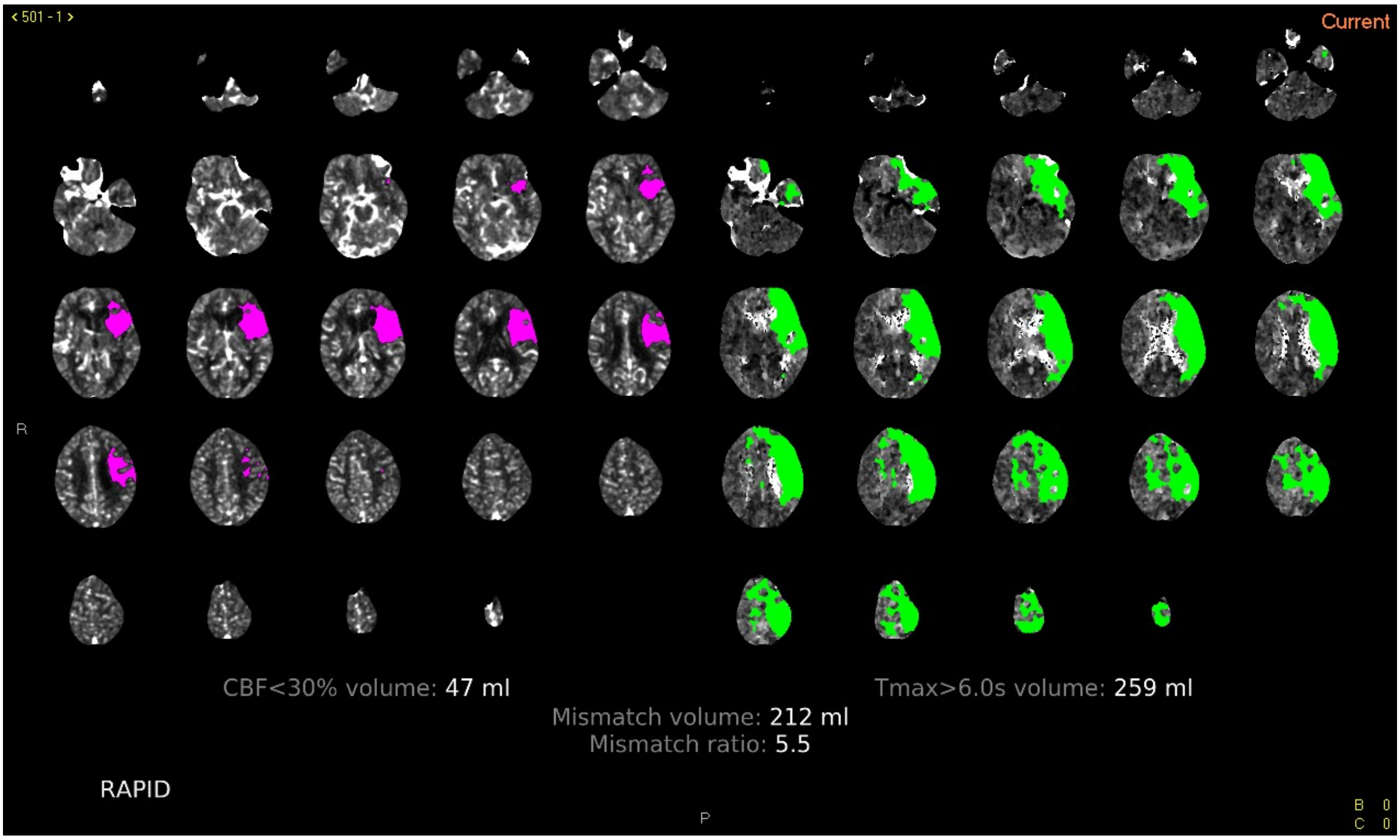

Based on the clinical and radiologic details will you recommend endovascular thrombectomy for this patient?

- ◯ Yes
- ◯ No

Case 38: 48

Case Details
Demographics 82-year-old male
Occlusion Left M1
NIHSS 14
Last known well time 08:00
Time of CT scout film (Start of CT) 22:02

Non-contrast CT

What is the ASPECTS of this scan? __________________________________

Single Phase CTA

What is the single-phase collateral score for this scan?

- ◯ Collateral Score 0: Absence of vessels on CTA distal to the occlusion
- ◯ Collateral Score 1: Collateral supply filling < or equal to 50% but >0% of the occluded MCA territory.
- ◯ Collateral Score 2: Collateral supply filling >50% but < 100% of the occluded MCA territory
- ◯ Collateral Score 3: 100% collateral supply of the occluded MCA territory

Based on the clinical and radiologic details will you recommend endovascular thrombectomy for this patient?

- ◯ Yes
- ◯ No

Case 39: 56

Case Details
Demographics 51-year-old male
Occlusion Left M1
NIHSS 7
Last known well time 05:30
Time of CT scout film (Start of CT) 18:38

Non-contrast CT

What is the ASPECTS of this scan? ________________________

Single Phase CTA

What is the single-phase collateral score for this scan?

- ◯ Collateral Score 0: Absence of vessels on CTA distal to the occlusion
- ◯ Collateral Score 1: Collateral supply filling < or equal to 50% but >0% of the occluded MCA territory.
- ◯ Collateral Score 2: Collateral supply filling >50% but < 100% of the occluded MCA territory
- ◯ Collateral Score 3: 100% collateral supply of the occluded MCA territory

Based on the clinical and radiologic details will you recommend endovascular thrombectomy for this patient?

- ◯ Yes
- ◯ No

Case 40: 60

Case Details
Demographics 74-year-old male
Occlusion Left carotid termination
NIHSS 22
Last known well time 23:00 The night prior
Time of CT scout film (Start of CT) 09:47 of the current day

Non-contrast CT

What is the ASPECTS of this scan? __________________________________

Single Phase CTA

What is the single-phase collateral score for this scan?

- ◯ Collateral Score 0: Absence of vessels on CTA distal to the occlusion
- ◯ Collateral Score 1: Collateral supply filling < or equal to 50% but >0% of the occluded MCA territory.
- ◯ Collateral Score 2: Collateral supply filling >50% but < 100% of the occluded MCA territory
- ◯ Collateral Score 3: 100% collateral supply of the occluded MCA territory

Based on the clinical and radiologic details will you recommend endovascular thrombectomy for this patient?

- ◯ Yes
- ◯ No

Case 41: 14

Case Details
Demographics 79-year-old female
Occlusion Left carotid termination
NIHSS 24
Last known well time 04:30
Time of CT scout film (Start of CT) 11:27

Non-contrast CT

What is the ASPECTS of this scan? ______________________

Single Phase CTA

What is the single-phase collateral score for this scan?

- ◯ Collateral Score 0: Absence of vessels on CTA is consistent with
- ◯ Collateral Score 1: Collateral supply filling < or equal to 50% but >0% of the occluded MCA territory.
- ◯ Collateral Score 2: Collateral supply filling >50% but < 100% of the occluded MCA territory
- ◯ Collateral Score 3: 100% collateral supply of the occluded MCA territory

---

Based on the clinical and radiologic details will you recommend endovascular thrombectomy for this patient?

- ◯ Yes
- ◯ No

Case 42: 45

Case Details
Demographics 81-year-old female
Occlusion Left M1
NIHSS 12
Last known well time 01:00
Time of CT scout film (Start of CT) 11:40

Non-contrast CT

What is the ASPECTS of this scan?

____

Single Phase CTA

What is the single-phase collateral score for this scan?

- ◯ Collateral Score 0: Absence of vessels on CTA distal to the occlusion
- ◯ Collateral Score 1: Collateral supply filling < or equal to 50% but >0% of the occluded MCA territory.
- ◯ Collateral Score 2: Collateral supply filling >50% but < 100% of the occluded MCA territory
- ◯ Collateral Score 3: 100% collateral supply of the occluded MCA territory

Automate Perfusion Scan Results

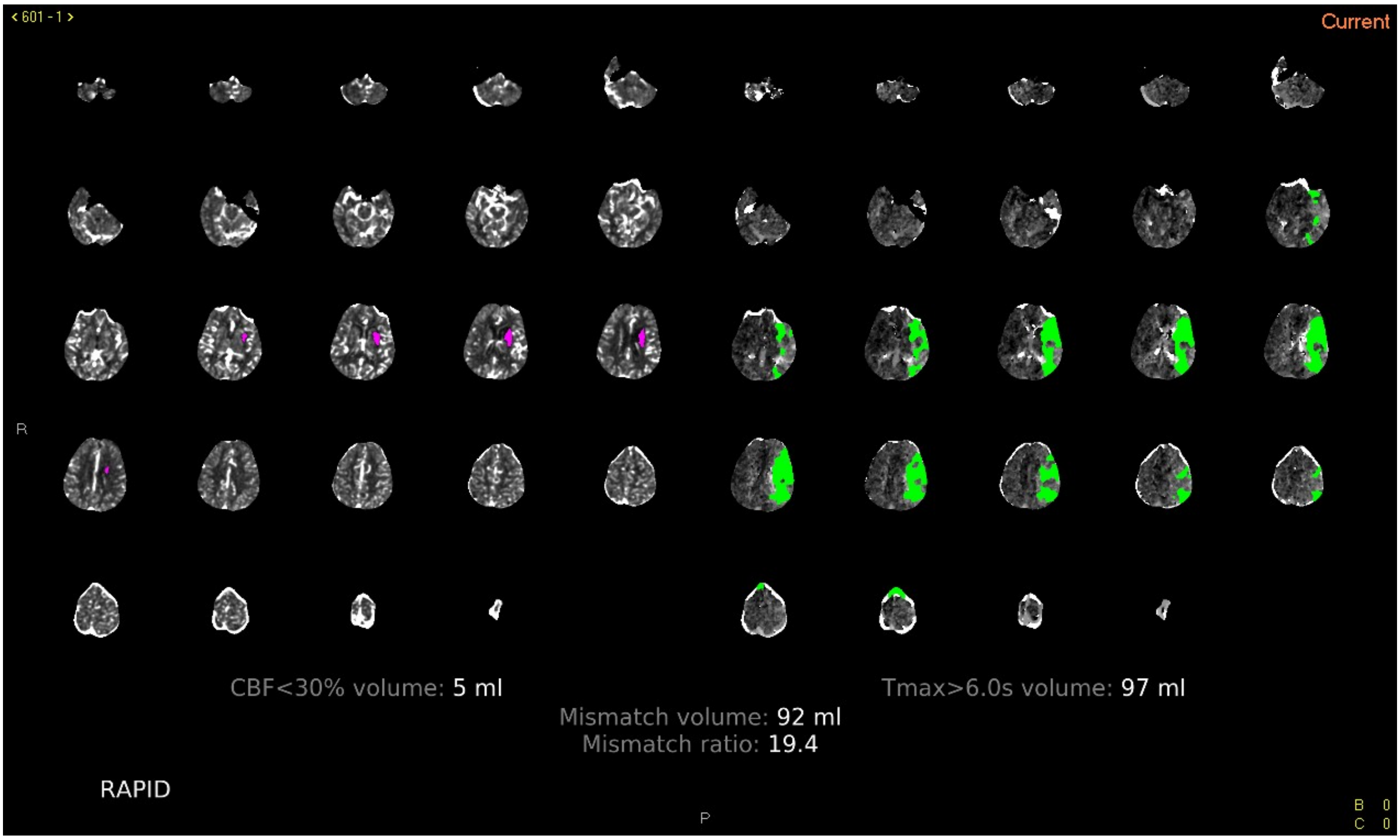

Based on the clinical and radiologic details will you recommend endovascular thrombectomy for this patient?

- ◯ Yes
- ◯ No

Case 43: 29

Case Details
Demographics 83-year-old female
Occlusion Right M1
NIHSS 6
Last known well time 23:00 The night prior
Time of CT scout film (Start of CT) 16:57 of the current day

Non-contrast CT

What is the ASPECTS of this scan? __________________________________

Single Phase CTA

What is the single-phase collateral score for this scan?

- ◯ Collateral Score 0: Absence of vessels on CTA distal to the occlusion
- ◯ Collateral Score 1: Collateral supply filling < or equal to 50% but >0% of the occluded MCA territory.
- ◯ Collateral Score 2: Collateral supply filling >50% but < 100% of the occluded MCA territory
- ◯ Collateral Score 3: 100% collateral supply of the occluded MCA territory

Automate Perfusion Scan Results

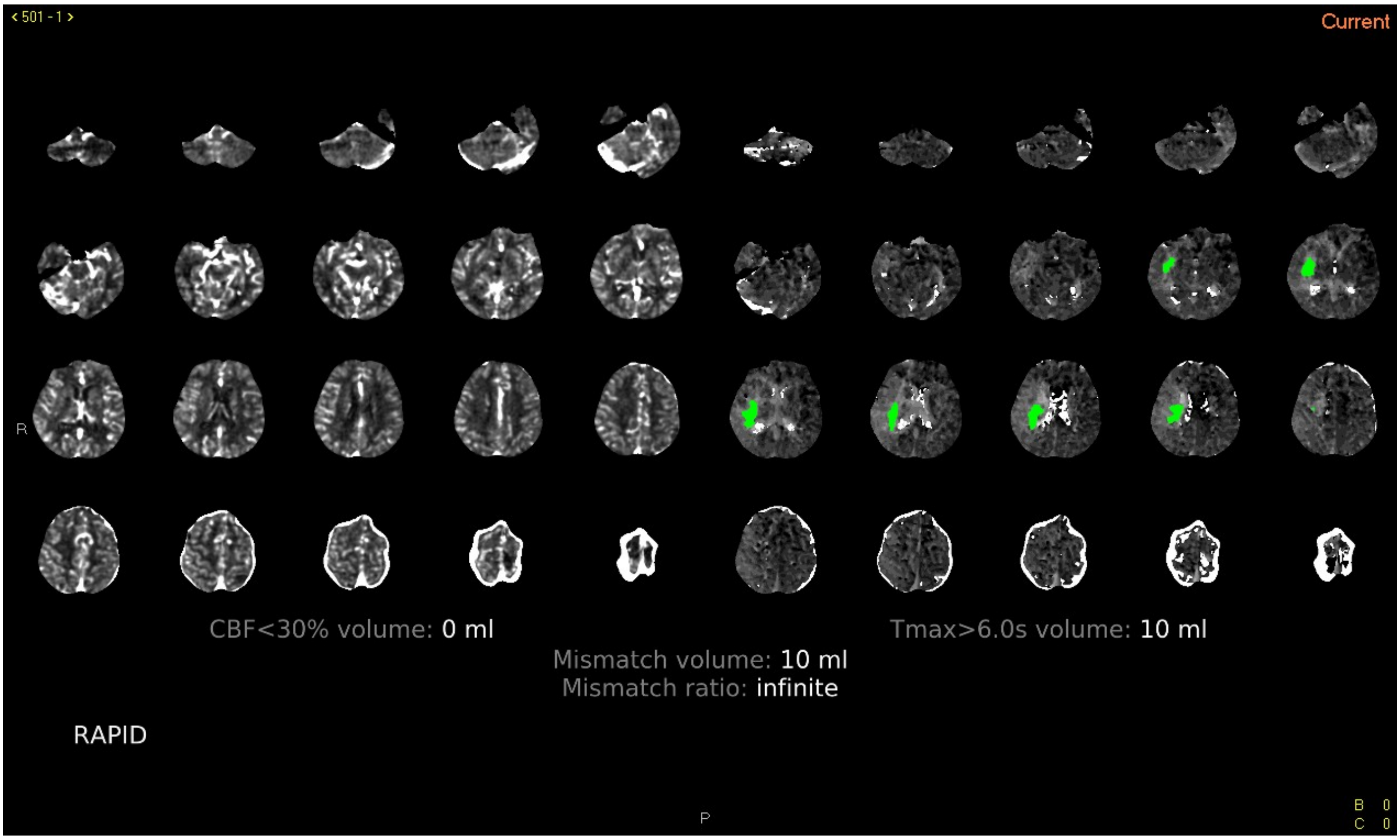

Based on the clinical and radiologic details will you recommend endovascular thrombectomy for this patient?

- ◯ Yes
- ◯ No

Case 44: 38

Case Details
Demographics 75-year-old male
Occlusion Right tandem occlusion
NIHSS 24
Last known well time 18:00 The night prior
Time of CT scout film (Start of CT) 13:00 of the current day

Non-contrast CT

What is the ASPECTS of this scan?

____

Single Phase CTA

What is the single-phase collateral score for this scan?

- ◯ Collateral Score 0: Absence of vessels on CTA distal to the occlusion
- ◯ Collateral Score 1: Collateral supply filling < or equal to 50% but >0% of the occluded MCA territory.
- ◯ Collateral Score 2: Collateral supply filling >50% but < 100% of the occluded MCA territory
- ◯ Collateral Score 3: 100% collateral supply of the occluded MCA territory

Based on the clinical and radiologic details will you recommend endovascular thrombectomy for this patient?

- ◯ Yes
- ◯ No

Case 45: 34

Case Details
Demographics 70-year-old male
Occlusion Left tandem occlusion
NIHSS 8
Last known well time 13:05 The day prior
Time of CT scout film (Start of CT) 00:36 of the current day

Non-contrast CT

What is the ASPECTS of this scan? __________________________________

Single Phase CTA

What is the single-phase collateral score for this scan?

- ◯ Collateral Score 0: Absence of vessels on CTA distal to the occlusion
- ◯ Collateral Score 1: Collateral supply filling < or equal to 50% but >0% of the occluded MCA territory.
- ◯ Collateral Score 2: Collateral supply filling >50% but < 100% of the occluded MCA territory
- ◯ Collateral Score 3: 100% collateral supply of the occluded MCA territory

Based on the clinical and radiologic details will you recommend endovascular thrombectomy for this patient?

- ◯ Yes
- ◯ No

Case 46: 09

Case Details
Demographics 39-year-old female
Occlusion Left M1
NIHSS 9
Last known well time 22:00 of the previous day
Time of CT scout film (Start of CT) 13:12 of the current day

Non-contrast CT

What is the ASPECTS of this scan? ______________________

Single Phase CTA

What is the single-phase collateral score for this scan?

- ◯ Collateral Score 0: Absence of vessels on CTA distal to the occlusion
- ◯ Collateral Score 1: Collateral supply filling < or equal to 50% but >0% of the occluded MCA territory.
- ◯ Collateral Score 2: Collateral supply filling >50% but < 100% of the occluded MCA territory
- ◯ Collateral Score 3: 100% collateral supply of the occluded MCA territory

Automate Perfusion Scan Results

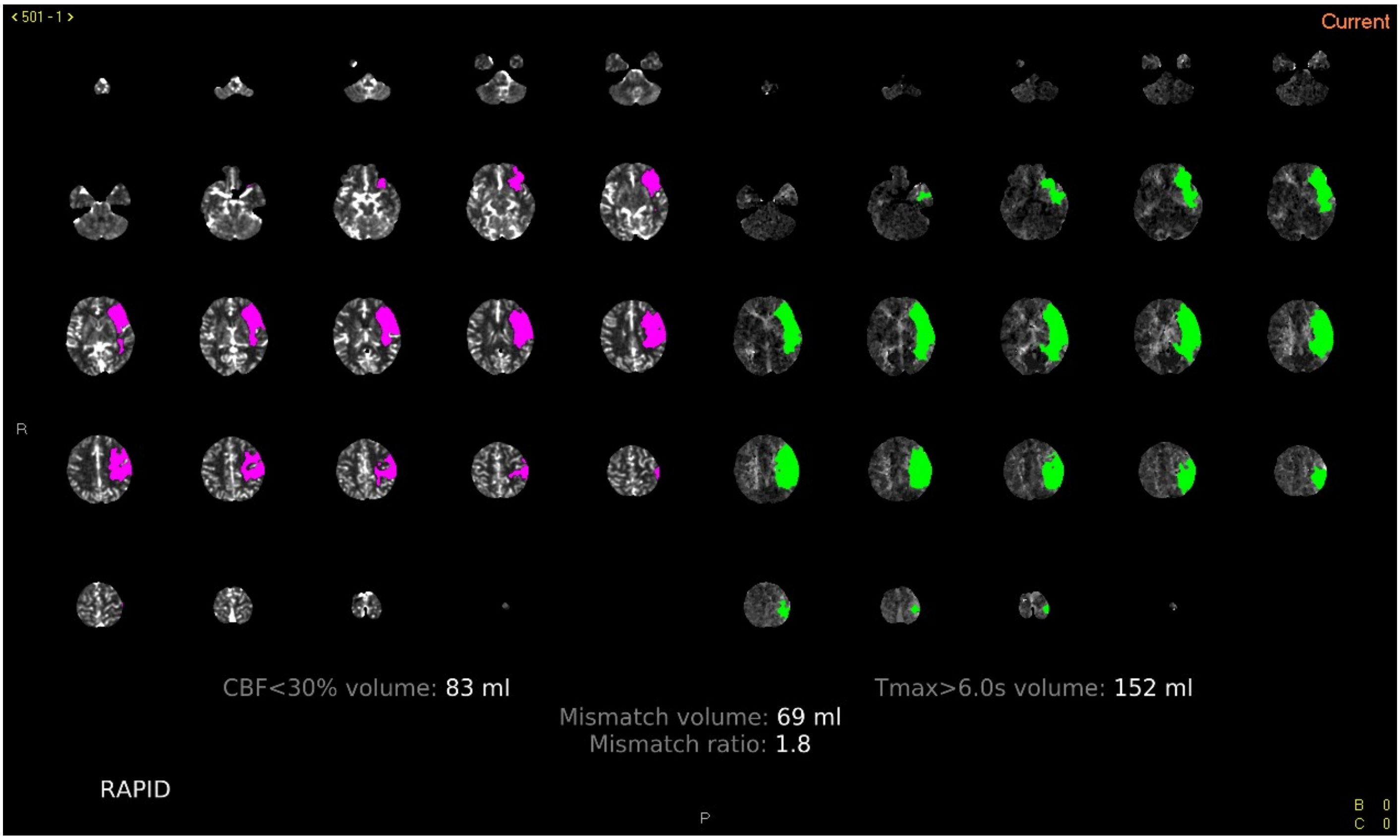

Based on the clinical and radiologic details will you recommend endovascular thrombectomy for this patient?

- ◯ Yes
- ◯ No

Case 47: 35

Case Details
Demographics 76-year-old male
Occlusion Right M1
NIHSS 17
Last known well time 23:00 The night prior
Time of CT scout film (Start of CT) 06:07 of the current day

Non-contrast CT

What is the ASPECTS of this scan? ______________________

Single Phase CTA

What is the single-phase collateral score for this scan?

- ◯ Collateral Score 0: Absence of vessels on CTA distal to the occlusion
- ◯ Collateral Score 1: Collateral supply filling < or equal to 50% but >0% of the occluded MCA territory.
- ◯ Collateral Score 2: Collateral supply filling >50% but < 100% of the occluded MCA territory
- ◯ Collateral Score 3: 100% collateral supply of the occluded MCA territory

Automate Perfusion Scan Results

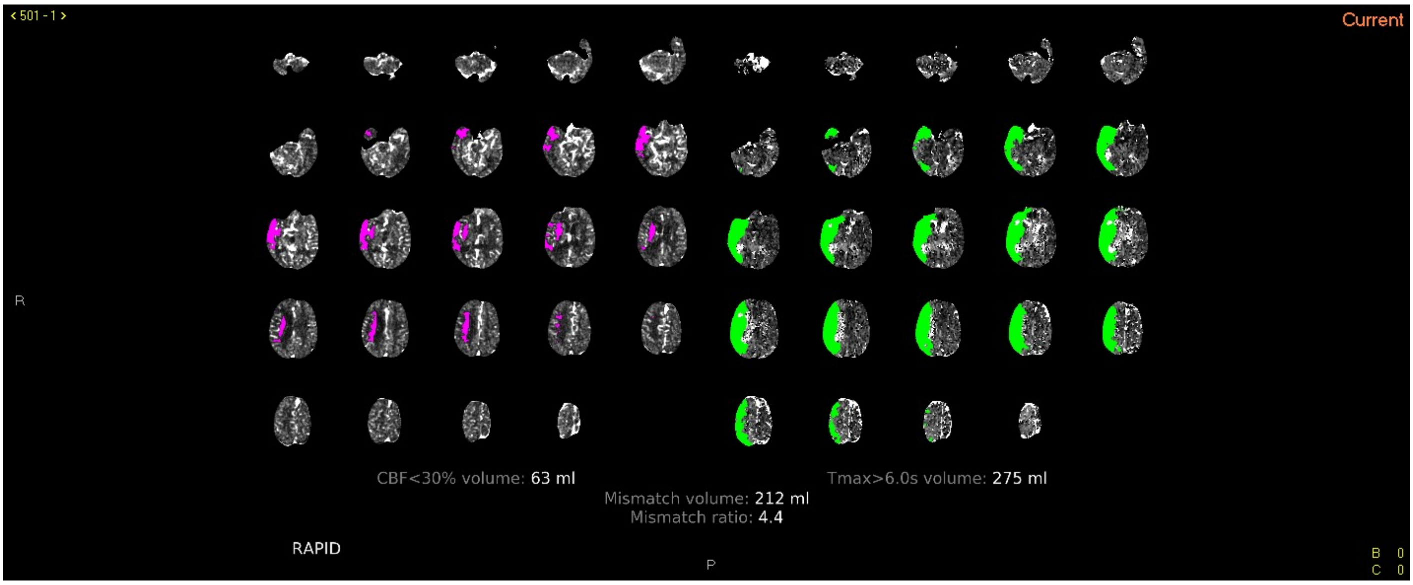

Based on the clinical and radiologic details will you recommend endovascular thrombectomy for this patient?

- ◯ Yes
- ◯ No

Case 48: 02

Case Details
Demographics 87-year-old female
Occlusion Left carotid termination
NIHSS 27
Last known well time 21:10 The night prior
Time of CT scout film (Start of CT) 04:07 of the current day

Non-contrast CT

What is the ASPECTS of this scan? ________________________________

Single Phase CTA

What is the single-phase collateral score for this scan?

- ◯ Collateral Score 0: Absence of vessels on CTA distal to the occlusion
- ◯ Collateral Score 1: Collateral supply filling < or equal to 50% but >0% of the occluded MCA territory.
- ◯ Collateral Score 2: Collateral supply filling >50% but < 100% of the occluded MCA territory
- ◯ Collateral Score 3: 100% collateral supply of the occluded MCA territory

Based on the clinical and radiologic details will you recommend endovascular thrombectomy for this patient?

- ◯ Yes
- ◯ No

Case 49: 46

Case Details
Demographics 81-year-old female
Occlusion Left M1
NIHSS 12
Last known well time 01:00
Time of CT scout film (Start of CT) 11:40

Non-contrast CT

What is the ASPECTS of this scan? ____________________

Single Phase CTA

What is the single-phase collateral score for this scan?

- ◯ Collateral Score 0: Absence of vessels on CTA distal to the occlusion
- ◯ Collateral Score 1: Collateral supply filling < or equal to 50% but >0% of the occluded MCA territory.
- ◯ Collateral Score 2: Collateral supply filling >50% but < 100% of the occluded MCA territory
- ◯ Collateral Score 3: 100% collateral supply of the occluded MCA territory

Based on the clinical and radiologic details will you recommend endovascular thrombectomy for this patient?

- ◯ Yes
- ◯ No

Case 50: 07

Case Details
Demographics 41-year-old male
Occlusion Left M1
NIHSS 25
Last known well time 23:00 The night prior
Time of CT scout film (Start of CT) 06:10 of the current day

Non-contrast CT

What is the ASPECTS of this scan?

____

Single Phase CTA

What is the single-phase collateral score for this scan?

- ◯ Collateral Score 0: Absence of vessels on CTA distal to the occlusion
- ◯ Collateral Score 1: Collateral supply filling < or equal to 50% but >0% of the occluded MCA territory.
- ◯ Collateral Score 2: Collateral supply filling >50% but < 100% of the occluded MCA territory
- ◯ Collateral Score 3: 100% collateral supply of the occluded MCA territory

Automate Perfusion Scan Results

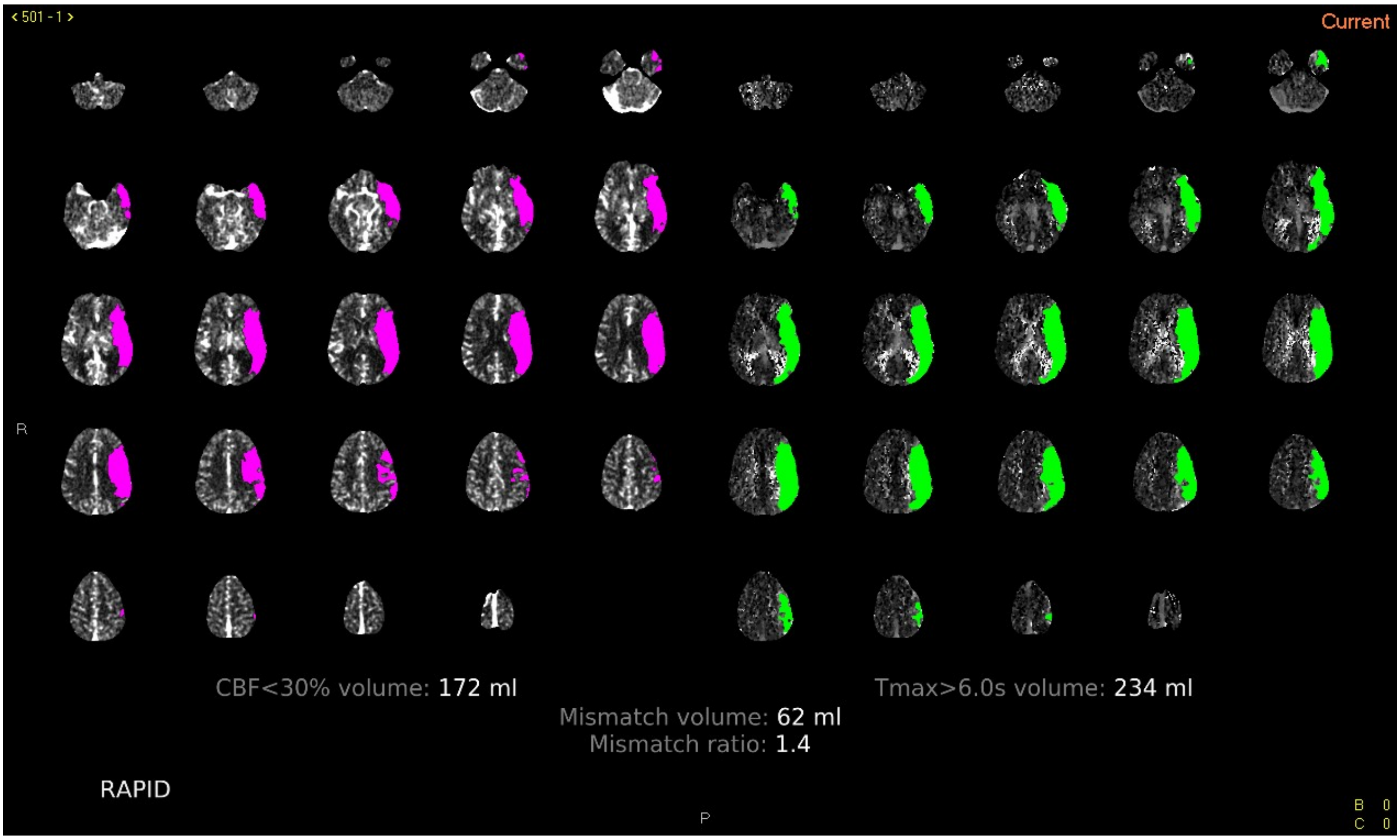

Based on the clinical and radiologic details will you recommend endovascular thrombectomy for this patient?

- ◯ Yes
- ◯ No

Case 51: 28

Case Details
Demographics 40-year-old female
Occlusion Left M1
NIHSS 8
Last known well time 23:30 The night prior
Time of CT scout film (Start of CT) 09:44 of the current day

Non-contrast CT

What is the ASPECTS of this scan? __________________________________

Single Phase CTA

What is the single-phase collateral score for this scan?

- ◯ Collateral Score 0: Absence of vessels on CTA distal to the occlusion
- ◯ Collateral Score 1: Collateral supply filling < or equal to 50% but >0% of the occluded MCA territory.
- ◯ Collateral Score 2: Collateral supply filling >50% but < 100% of the occluded MCA territory
- ◯ Collateral Score 3: 100% collateral supply of the occluded MCA territory

Based on the clinical and radiologic details will you recommend endovascular thrombectomy for this patient?

- ◯ Yes
- ◯ No

Case 52: 30

Case Details
Demographics 83-year-old female
Occlusion Right M1
NIHSS 6
Last known well time 23:00 The night prior
Time of CT scout film (Start of CT) 16:57 of the current day

Non-contrast CT

What is the ASPECTS of this scan? __________________________________

Single Phase CTA

What is the single-phase collateral score for this scan?

- ◯ Collateral Score 0: Absence of vessels on CTA distal to the occlusion
- ◯ Collateral Score 1: Collateral supply filling < or equal to 50% but >0% of the occluded MCA territory.
- ◯ Collateral Score 2: Collateral supply filling >50% but < 100% of the occluded MCA territory
- ◯ Collateral Score 3: 100% collateral supply of the occluded MCA territory

Based on the clinical and radiologic details will you recommend endovascular thrombectomy for this patient?

- ◯ Yes
- ◯ No

Case 53: 26

Case Details
Demographics 92-year-old female
Occlusion Left M1
NIHSS 21
Last known well time 15:00
Time of CT scout film (Start of CT) 22:50

Non-contrast CT

What is the ASPECTS of this scan? __________________________________

Single Phase CTA

What is the single-phase collateral score for this scan?

- ◯ Collateral Score 0: Absence of vessels on CTA distal to the occlusion
- ◯ Collateral Score 1: Collateral supply filling < or equal to 50% but >0% of the occluded MCA territory.
- ◯ Collateral Score 2: Collateral supply filling >50% but < 100% of the occluded MCA territory
- ◯ Collateral Score 3: 100% collateral supply of the occluded MCA territory

Based on the clinical and radiologic details will you recommend endovascular thrombectomy for this patient?

- ◯ Yes
- ◯ No

Case 54: 08

Case Details
Demographics 41-year-old male
Occlusion Left M1
NIHSS 25
Last known well time 23:00 The night prior
Time of CT scout film (Start of CT) 06:10 of the current day

Non-contrast CT

What is the ASPECTS of this scan? __________________________________

Single Phase CTA

What is the single-phase collateral score for this scan?

- ◯ Collateral Score 0: Absence of vessels on CTA distal to the occlusion
- ◯ Collateral Score 1: Collateral supply filling < or equal to 50% but >0% of the occluded MCA territory.
- ◯ Collateral Score 2: Collateral supply filling >50% but < 100% of the occluded MCA territory
- ◯ Collateral Score 3: 100% collateral supply of the occluded MCA territory

Based on the clinical and radiologic details will you recommend endovascular thrombectomy for this patient?

- ◯ Yes
- ◯ No

Case 55: 36

Case Details
Demographics 76-year-old male
Occlusion Right M1
NIHSS 17
Last known well time 23:00 The night prior
Time of CT scout film (Start of CT) 06:07 of the current day

Non-contrast CT

What is the ASPECTS of this scan? ______________________

Single Phase CTA

What is the single-phase collateral score for this scan?

- ◯ Collateral Score 0: Absence of vessels on CTA distal to the occlusion
- ◯ Collateral Score 1: Collateral supply filling < or equal to 50% but >0% of the occluded MCA territory.
- ◯ Collateral Score 2: Collateral supply filling >50% but < 100% of the occluded MCA territory
- ◯ Collateral Score 3: 100% collateral supply of the occluded MCA territory

Based on the clinical and radiologic details will you recommend endovascular thrombectomy for this patient?

- ◯ Yes
- ◯ No

Case 56: 57

Case Details
Demographics 71-year-old male
Occlusion Right M1
NIHSS 19
Last known well time 02:30
Time of CT scout film (Start of CT) 11:23

Non-contrast CT

What is the ASPECTS of this scan? ________________________

Single Phase CTA

What is the single-phase collateral score for this scan?

- ◯ Collateral Score 0: Absence of vessels on CTA distal to the occlusion
- ◯ Collateral Score 1: Collateral supply filling < or equal to 50% but >0% of the occluded MCA territory.
- ◯ Collateral Score 2: Collateral supply filling >50% but < 100% of the occluded MCA territory
- ◯ Collateral Score 3: 100% collateral supply of the occluded MCA territory

Automate Perfusion Scan Results

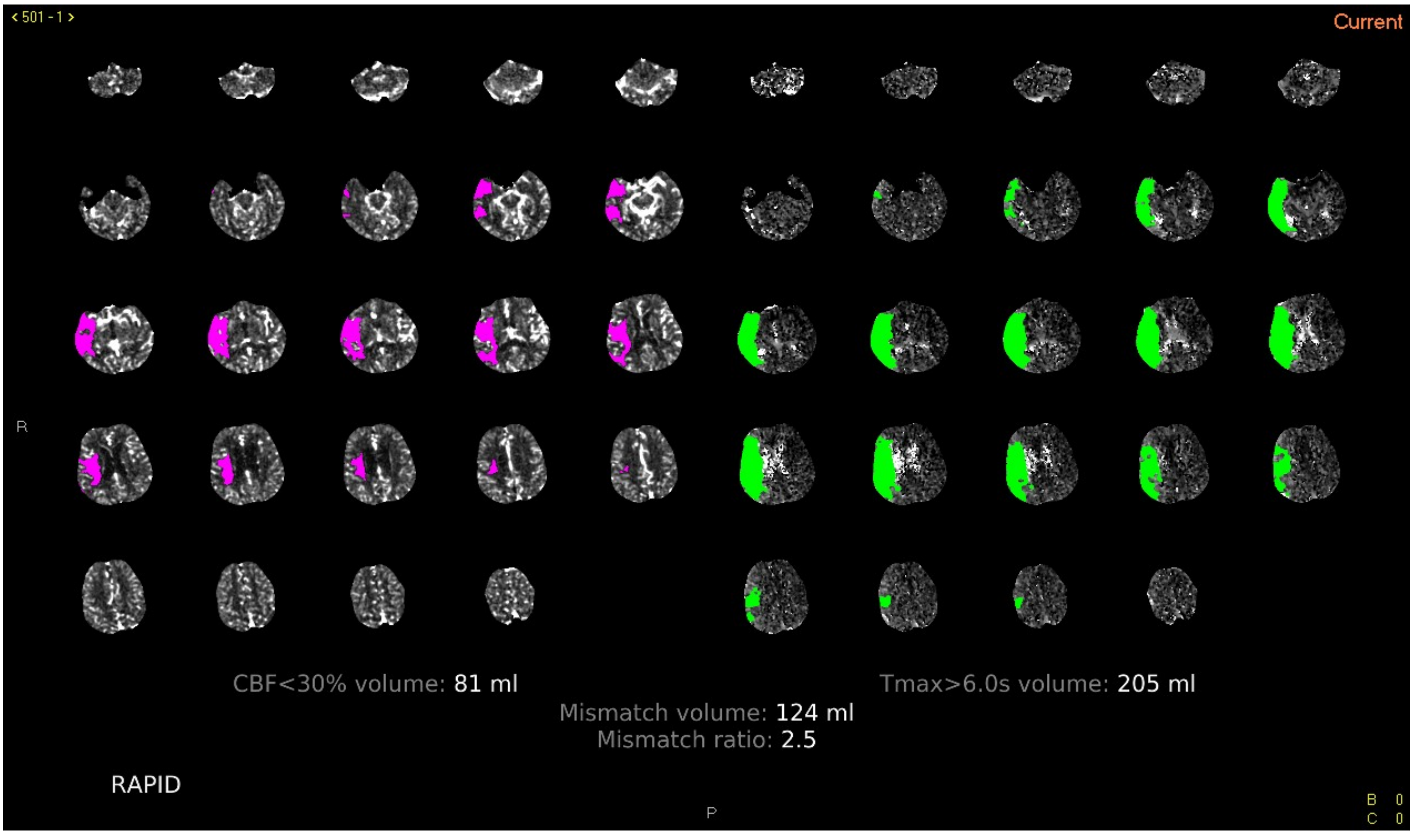

Based on the clinical and radiologic details will you recommend endovascular thrombectomy for this patient?

- ◯ Yes
- ◯ No

Case 57: 55

Case Details
Demographics 51-year-old male
Occlusion Left M1
NIHSS 7
Last known well time 05:30
Time of CT scout film (Start of CT) 18:38

Non-contrast CT

What is the ASPECTS of this scan? ________________________________

Single Phase CTA

What is the single-phase collateral score for this scan?

- ◯ Collateral Score 0: Absence of vessels on CTA distal to the occlusion
- ◯ Collateral Score 1: Collateral supply filling < or equal to 50% but >0% of the occluded MCA territory.
- ◯ Collateral Score 2: Collateral supply filling >50% but < 100% of the occluded MCA territory
- ◯ Collateral Score 3: 100% collateral supply of the occluded MCA territory

Automate Perfusion Scan Results

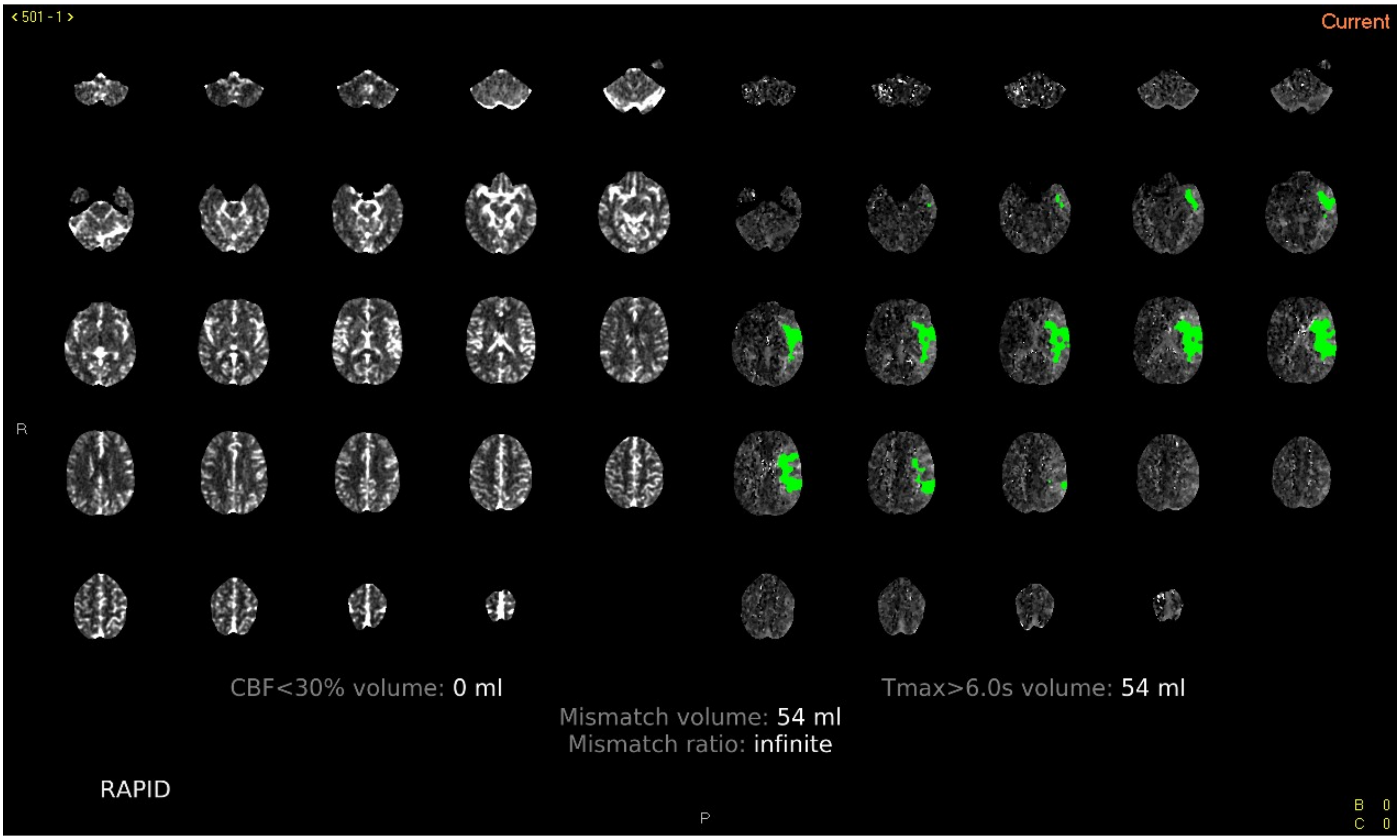

Based on the clinical and radiologic details will you recommend endovascular thrombectomy for this patient?

- ◯ Yes
- ◯ No

Case 58: 03

Case Details
Demographics 82-year-old male
Occlusion Right M1
NIHSS 14
Last known well time 00:10
Time of CT scout film (Start of CT) 15:13

Non-contrast CT

What is the ASPECTS of this scan? ____________________

Single Phase CTA

What is the single-phase collateral score for this scan?

- ◯ Collateral Score 0: Absence of vessels on CTA distal to the occlusion
- ◯ Collateral Score 1: Collateral supply filling < or equal to 50% but >0% of the occluded MCA territory.
- ◯ Collateral Score 2: Collateral supply filling >50% but < 100% of the occluded MCA territory
- ◯ Collateral Score 3: 100% collateral supply of the occluded MCA territory

Automate Perfusion Scan Results

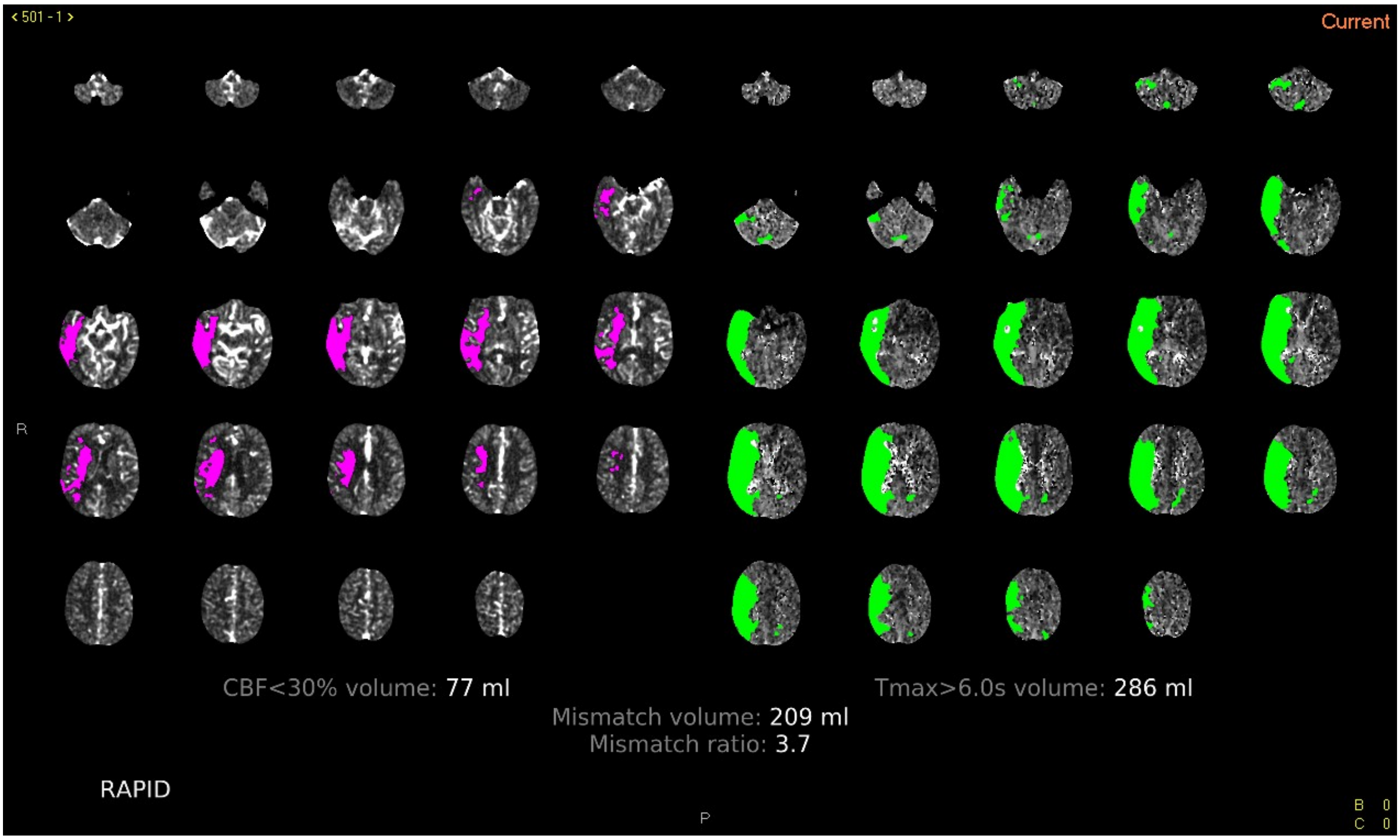

Based on the clinical and radiologic details will you recommend endovascular thrombectomy for this patient?

- ◯ Yes
- ◯ No

Case 59: 53

Case Details
Demographics 81-year-old female
Occlusion Left carotid termination
NIHSS 18
Last known well time 21:30 The night prior
Time of CT scout film (Start of CT) 05:58 of the current day

Non-contrast CT

What is the ASPECTS of this scan? __________________________________

Single Phase CTA

What is the single-phase collateral score for this scan?

- ◯ Collateral Score 0: Absence of vessels on CTA distal to the occlusion
- ◯ Collateral Score 1: Collateral supply filling < or equal to 50% but >0% of the occluded MCA territory.
- ◯ Collateral Score 2: Collateral supply filling >50% but < 100% of the occluded MCA territory
- ◯ Collateral Score 3: 100% collateral supply of the occluded MCA territory

Automate Perfusion Scan Results

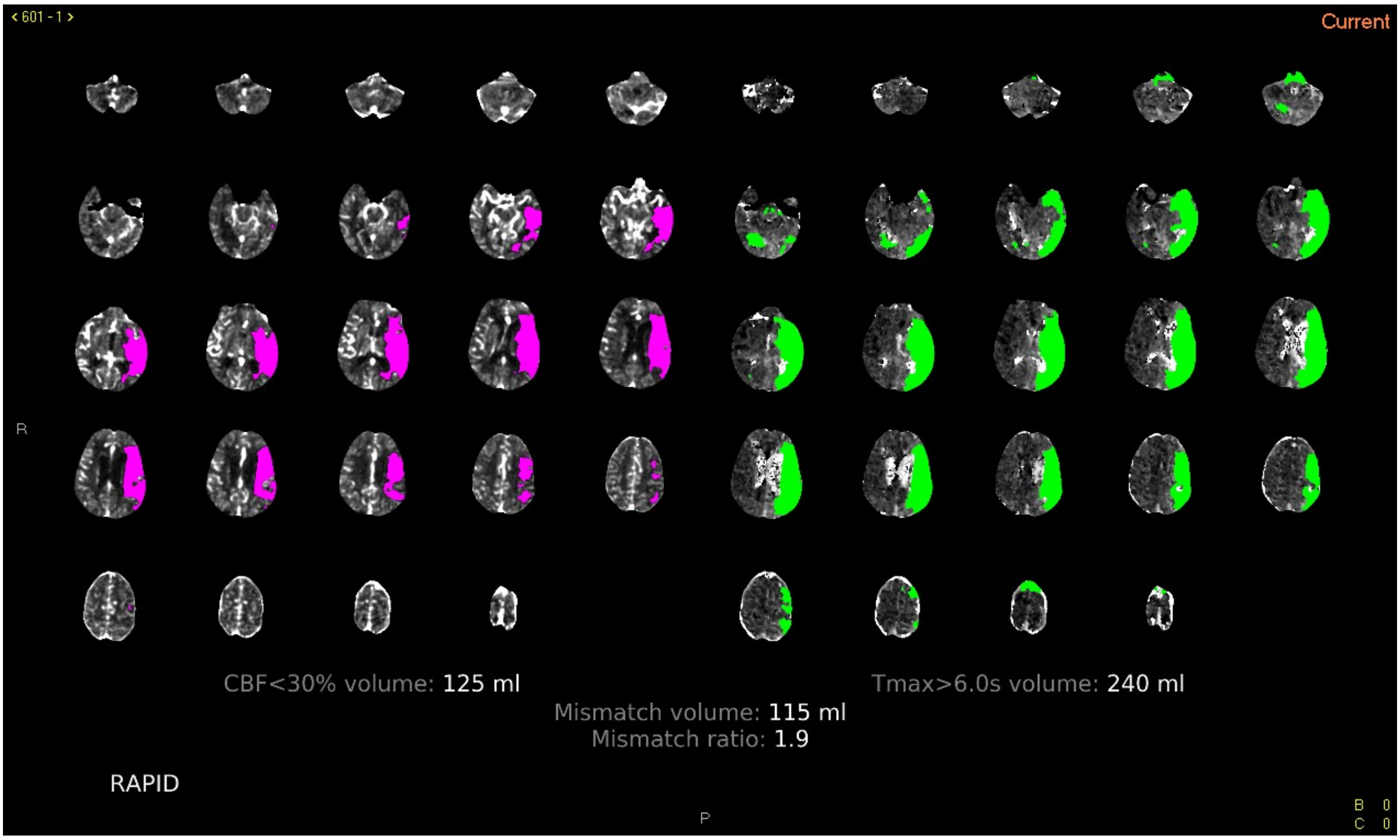

Based on the clinical and radiologic details will you recommend endovascular thrombectomy for this patient?

- ◯ Yes
- ◯ No

Case 60: 25

Case Details
Demographics 92-year-old female
Occlusion Left M1
NIHSS 21
Last known well time 15:00
Time of CT scout film (Start of CT) 22:50

Non-contrast CT

What is the ASPECTS of this scan?

____

Single Phase CTA

What is the single-phase collateral score for this scan?

- ◯ Collateral Score 0: Absence of vessels on CTA distal to the occlusion
- ◯ Collateral Score 1: Collateral supply filling < or equal to 50% but >0% of the occluded MCA territory.
- ◯ Collateral Score 2: Collateral supply filling >50% but < 100% of the occluded MCA territory
- ◯ Collateral Score 3: 100% collateral supply of the occluded MCA territory

Automate Perfusion Scan Results

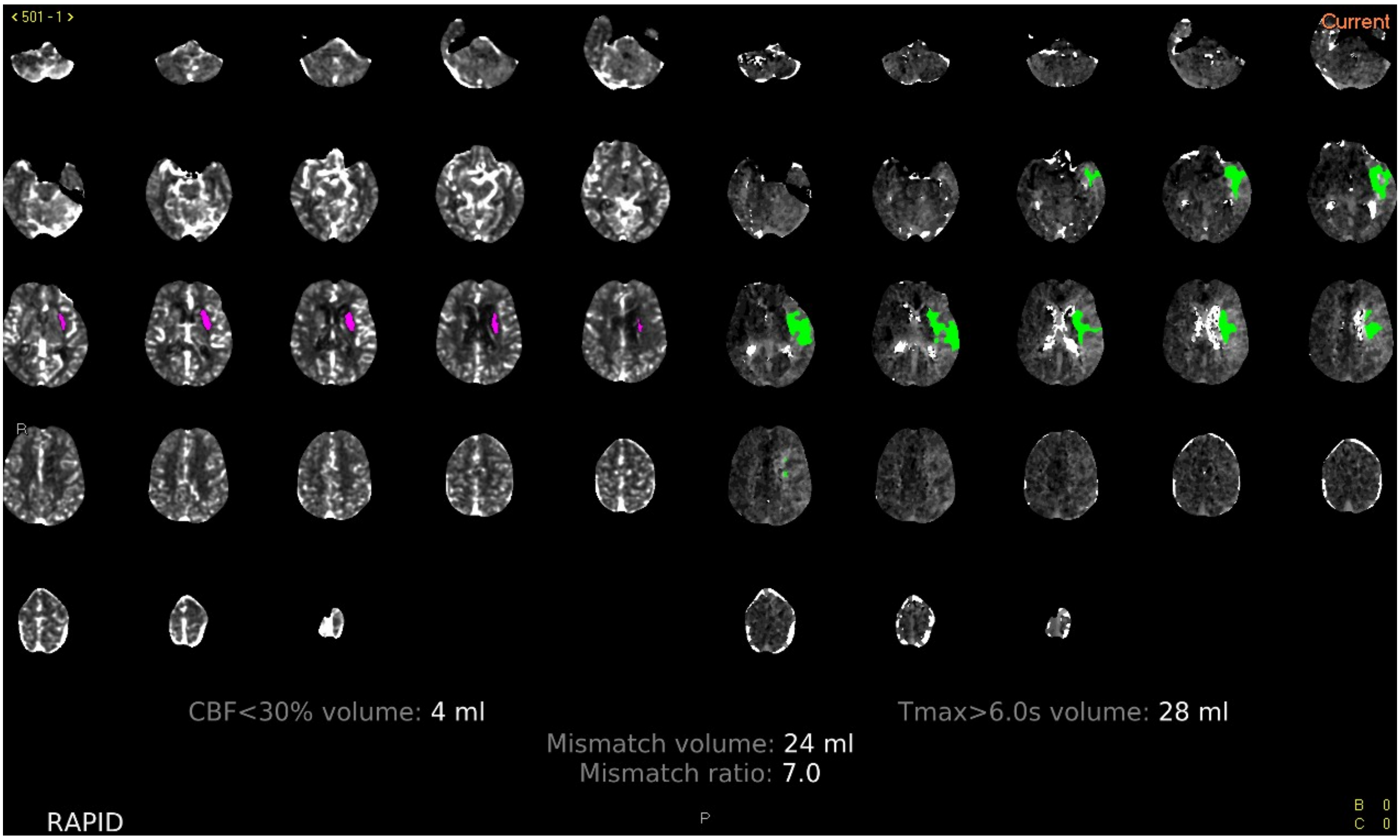

Based on the clinical and radiologic details will you recommend endovascular thrombectomy for this patient?

- ◯ Yes
- ◯ No
