# Supplementary material for: Clinical Uncertainty in Large Vessel Occlusion ischemic stroke (CULVO): Does automated perfusion scanning make a difference? Protocol of an intrarater and interrater agreement study
Source: PLoS One. 2024 Jan 30;19(1):e0297520. doi: 10.1371/journal.pone.0297520 (PMC10826946; doi:10.1371/journal.pone.0297520)
Supplement: S1 File — (PDF) [file pone.0297520.s002.pdf]

Research Ethics Office
Telephone: (416) 864-6060 Ext. 42557
Facsimile: (416) 864-6043
E-mail: Dharmista.Patel@unityhealth.to

October 21, 2022

Dr. Vitor Pereira,
Department of Surgery, Division of Neurosurgery,
St. Michael's Hospital

Dear Dr. Pereira,

Re: REB# 22-186[C] - Clinical uncertainty in large vessel occlusion ischemic stroke: Does automated perfusion scanning make a difference? An intrarater and interrater agreement study

**REB APPROVAL:**	**Original Approval Date**	**October 21, 2022**
	**Annual/Interval Review Date**	**October 21, 2023**

Thank you for your application submitted on **08 August, 2022**. The above noted study has been reviewed through a delegated process (not by Full Board review). The views of the Unity Health Toronto Research Ethics Board (REB) have been documented and resolved. Please note that no member of the REB associated with this study was involved in its review or approval.

The REB approves the study as it is found to comply with relevant research ethics guidelines, as well as the Ontario Personal Health Information Protection Act (PHIPA), 2004. The REB hereby issues approval for the above named study for a period of 12 months from the date of this letter. Continuation beyond that date will require further review of REB approval.

In addition, the following documents have been reviewed and are hereby approved:

1. Protocol - ver: 2 07-Sep-2022
2. Consent Form - ver: 19-Sep-2022
3. Letter of Invitation - ver: 19-Sep-2022
4. Email - REDCap link email - ver: 26-Aug-2022
5. Debriefing letter - ver: 19-Sep-2022

Furthermore, the following documents have been received and are acknowledged:

1. Master Linking Log - Patient ver: 26-Aug-2022
2. Master Linking Log - Rater ver: 03-Aug-2022
3. Data Collection Form - Patient ver: 26-Aug-2022
4. Data Collection Form - Rater ver: 26-Aug-2022
5. Data Collection Form - Rater Responses ver: 19-Sep-2022
6. Sample REDCap Format ver: 07-Sep-2022

Please note that all clinical research studies are required to comply with the institution's guidelines related to on-site and in-person research. This REB approval letter does not override any restrictions covered in those guidelines.

During the course of this investigation, any significant deviations from the approved protocol and/or unanticipated developments or significant adverse events should immediately be brought to the attention of the REB. Please note that shared electronic health systems such as ConnectingOntario, PRO, RM&R, OLIS, HDIRS, eCHN, DPV and IAR do not permit access for research purposes.

For all interventional studies, the study sponsor is required to register the study in a publicly accessible registry prior to enrollment of the first study participant. The study sponsor is also required to ensure that the trial record is updated in a timely manner and the summary results are posted within the required timelines.

All institutional approvals **must be** coordinated and approved through the Office of Research Administration (ORA) prior to initiation of this research. If a Clinical Trial Agreement is required, it must be reviewed and approved by the ORA, prior to commencing any study related activities.

All investigational drug product dispensing must be coordinated through the Research Pharmacy.

REB operates in compliance with the Tri-Council Policy Statement Ethical Conduct for Research Involving Humans, the Ontario Personal Health Information Protection Act, 2004, and ICH Good Clinical Practice Consolidated Guideline E6, Health Canada Part C Division 5 of the Food and Drug Regulations, Part 4 of the Natural Health Product Regulations, and the Medical Devices regulations. Furthermore, all investigational drug trials at Unity Health Toronto are conducted by Qualified Investigators (as defined in the latter document).

With best wishes

☐ David Mazer, MD, Chair, REB ☐ Michael Szego, PhD, Vice Chair, REB
☒ Melanie Tsang, MD, Vice Chair, REB ☐ Zoe von Aesch, MD, Vice Chair, REB

DM/MS/MT/ZVA/al
